# Identification of the informational and supportive needs of patients diagnosed with inflammatory bowel disease: a scoping review

**DOI:** 10.3389/fpsyg.2023.1055449

**Published:** 2023-05-11

**Authors:** Narges Norouzkhani, Mahbobeh Faramarzi, Sara Ghodousi Moghadam, Mohammad Amin Karimi, Javad Shokri Shirvani, Ali Bahari, Mahdie ShojaeiBaghini, Saeid Eslami, Hamed Tabesh

**Affiliations:** ^1^Department of Medical Informatics, Faculty of Medicine, Mashhad University of Medical Sciences, Mashhad, Iran; ^2^Fatemeh Zahra Infertility and Reproductive Health Research Center, Babol University of Medical Sciences, Babol, Iran; ^3^Technology, Neyshabur University of Medical Sciences, Neyshabur, Iran; ^4^School of Medicine, Shahid Beheshti University of Medical Sciences, Tehran, Iran; ^5^Department of Internal Medicine, Babol University of Medical Sciences, Babol, Iran; ^6^Department of Internal Medicine, Faculty of Medicine, Mashhad University of Medical Sciences, Mashhad, Iran; ^7^Medical Informatics Research Center, Institute for Futures Studies in Health, Kerman University of Medical Sciences, Kerman, Iran; ^8^Pharmaceutical Research Center, Mashhad University of Medical Sciences, Mashhad, Iran; ^9^Department of Medical Informatics, Amsterdam UMC, University of Amsterdam, Amsterdam, Netherlands

**Keywords:** inflammatory bowel diseases, Needs Assessment, informational need, Information Seeking Behavior, Consumer Health Information, supportive needs, psychological needs

## Abstract

**Background:**

Inflammatory Bowel Disease (IBD) affects the quality of life. Patient education and support needs are crucial components of comprehensive chronic illness care. The main purposes of this review were to (i) explore the informational and supportive needs of these patients to improve the quality of life in the existing literature and (ii) identify the gaps related to the needs of the patients in articles.

**Methods:**

The scoping review is based on the Daudt methodological framework, a modified version of Arksey and O'Malley. Electronic databases were extensively searched from January 01, 2000 to April 30, 2022. Four electronic databases (PubMed/Medline, CINAHL, APA PsycInfo, Psychology and Behavioral Sciences Collection, APA PsycArticles, and ProQuest) were searched using controlled vocabulary, and specific keywords. The searched terms were matched to each database. We manually searched two key journals, namely the Journal of Inflammatory Bowel Disease and the Journal of Crohn's and Colitis.

**Results:**

In the review, 75 studies on the assessment of the information and support needs of patients with IBD were reviewed. In this regard, 62 and 53 studies were regarding information needs and support needs, respectively. Most of the information needs of patients with IBD reported in the studies were related to diet needs, and educational needs were the most essential support needs.

**Conclusions:**

Health policymakers and managers can develop care and educational programs related to this disease in health centers according to the needs of the patients. Health professionals, especially gastroenterologists, are the primary referral sources for information on patients. Therefore, gastroenterologists can take the lead in planning and educating the patients and sharing their decisions.

**Systematic review registration:**

OSF, https://doi.org/10.17605/OSF.IO/3MWGJ.

## 1. Introduction

Inflammatory bowel disease (IBD) occurs chronically and recurrently in the intestine (Cai et al., 2021). IBD has a relapsing-remitting pattern that is often unpredictable and causes inflammatory bowel flares (Fawson et al., 2021). Over 1 million people in the USA and 2.5 million in Europe are estimated to suffer from IBD.every year, newly diagnosed patients with IBD unremittingly increas the number of prevalent cases. This milieu sets up an epidemiological phenomenon called compounding prevalence; in other words, the number of patients with a disease such as IBD grows exponentially. The prevalence of IBD is, accordingly, expected to steadily ascend over the next decade in the Western world. The compounding of prevalence of IBD is a crucial concept in health-care delivery because healthcare systems might not be prepared for the significant increase in the burden of IBD (Kaplan, 2015).

Inflammatory bowel disease, Crohn's disease, and ulcerative colitis are considered idiopathic diseases affecting the gastrointestinal tract. These two diseases are usually considered together because of similarities, including gastrointestinal inflammation, waxing and waning severity and symptoms, and unknown etiology. However, they have separate symptoms and microscopic characteristics as well as patterns within the gastrointestinal tract (Thoreson and Cullen, 2007). Even though the pathogenesis of IBD is complicated, several studies have demonstrated that excessive interleukin (IL)-17 production is involved in the progression of IBD. Recently, research on IBD pathogenesis has focused on T helper (Th)17 cells, which secrete IL-17. It is well documented that Th17 inhibition can decrease the development of acute colitis by reducing inflammation.Additionally, innate lymphoid cells (ILCs) were recently discovered to be novel pathogenic effector lymphocytes in IBD (Lee et al., 2018).

CD onset occurs in a quarter of patients before adolescence and increases in the last four decades (Aloi and Cucchiara, 2022). The peak age of CD manifestations is between 20 and 30 years old, while the peak in UC is over 30 years old (Volpato et al., 2021). Although, the conditions are typically chronic and relapsing, UC can be managed by colon surgery (Strober et al., 2007).

In some cases, IBD requires several interventions and continuous monitoring and adaptation by patients and their caregivers, resulting in psychosocial suffering and hindering the patient's daily life (Graffigna et al., 2021). The patients experience decreased productivity, difficulty in studying, and feelings of depression or embarrassment associated with issues such as low self-esteem, impaired body image, finding accessible toilets, and difficulty in intimate relationships that severely impair the quality of life (Santos et al., 2020). It is still unclear how psychological factors can play a role in the development of people with IBD (Volpato et al., 2021). Furthermore, patients must use drugs for a long time and may need surgery. Hence, patients use ongoing education and guidelines related to this disease (Daher et al., 2019).

According to previous studies, most patients with IBD rely on gastroenterologists and the Internet as desirable and acceptable sources of information (Wong et al., 2012; Catalán-Serra et al., 2015). The care requirements of patients with IBD throughout childhood, adolescence, and adulthood were examined in a scoping review study (2021). According to the study, informational, medical, psychological, social, occupational, practical, future-related, and interactive demands were the most important among individuals with IBD (Volpato et al., 2021). More than 50% of the patients seek valid and reliable information about their disease, particularly drug treatment options (Karadag et al., 2020). Immune system-suppressing medications are frequently used in the current treatment of patients with IBD. Patients receiving these medications are more likely to catch illnesses, some of which can be shielded against by prompt vaccination (Long et al., 2013). According to a tertiary care center review of vaccination efforts, only 28% of patients with IBD obtained the annual influenza vaccine, and only 9% received the pneumococcal vaccine. This demonstrates that the patients lack accurate knowledge of vaccinations (Melmed et al., 2006). In addition, the patients feel abandoned by healthcare providers, since they pay more attention to treating the symptoms of the disease, and they give insufficient consideration to how the disease affects different aspects of an individual's life (Mapp, 2008).

The research found that patients lacked awareness of pregnancy, cancer, and the role of nutrition in disease management. They gave the most weight to details regarding causes of IBD, diet, symptoms, and novel treatments (Martin et al., 1992; Bernard et al., 2007). It is crucial to assess the needs of patients with IBD based on this information and the identified needs. Despite the high importance of the impact of IBD on patients' quality of life, there is no transparent and integrated information about information needs, supportive needs, and sources of information received from the patients. Hence, this scoping review aims to identify the informational and supportive needs of patients diagnosed with IBD.

## 2. Methods

The present scoping review is based on the Daudt methodological framework, a modified version of Arksey and O'Malley (2005) and Levac et al. (2010). In this study, six steps were completed.

### 2.1. Stage 1: identify the scope of objectives and inquiry

#### 2.1.1. Research questions

The purpose of our scoping review was to address the following research questions:

1. What are the informational needs of people with IBD?
2. What are the information resources needs of people with IBD?
3. What are the supportive needs of people with IBD?
4. What are the psychological needs of people with IBD?

#### 2.1.2. Study design

This scoping review was completed and conceptualized between October 2021 and July 2022 and was reviewed by experts experienced in methodological approaches. In this study, the needs of patients with IBD in different areas, including informational needs, information resources and supportive needs including psychological or emotional needs, physical needs, spiritual needs, informational needs, family-related needs, social needs, interpersonal or intimacy needs, practical needs, daily living needs, patient-clinician communication needs, cognitive needs, and support needs related to the future were operationalized. For this research, ethics approval was obtained from the Human Research Ethics Board (Mashhad University of Medical Sciences).

#### 2.1.3. Inclusion and exclusion criteria

Supplementary Table 1 presents the inclusion criteria and exclusion criteria. Patients with IBD and 18 years and older were included in this study. All types of studies were included in this scoping review. The focus of the study was on informational, supportive, and psychological needs. There were no restrictions on language or setting.

### 2.2. Stage 2: identify relevant studies

Initially, a consensus was achieved with a single strategy by comparing the two ones based on the inclusion and exclusion criteria used by two researchers (NN, MF).

Electronic databases were extensively searched from January 2000 to April 30, 2022, by two of the authors (NN, SGH). Four electronic databases (PubMed / Medline, CINAHL, APA PsycInfo, Psychology and Behavioral Sciences Collection, APA PsycArticles, and ProQuest/ProQuest One Academic) were searched using controlled vocabulary and specific keywords (Inflammatory Bowel Disease^*^, Crohn's Disease, Ulcerative Colitis, Needs Assessment^*^, Patient Need^*^, Patient Preference^*^, Patient Attitude^*^, Patient Expectation^*^, Educational Need^*^, Patient Education, Information^*^ Need^*^, Consumer Health Information, Information Seeking Behavior^*^, Information Seeking, Information Source, Supportive Need^*^, Support^*^ Need^*^, Psychological Need^*^, Mental Health Needs). Moreover, articles in the Journal of Inflammatory Bowel Disease and the Journal of Crohn's and Colitis were searched manually. The searched terms were combined using Boolean operators and matched to each database. Supplementary Table 2 provides a sample search strategy. A complete search in the references of retrieved articles was performed by one of the authors (NN) to find all related articles.

### 2.3. Stage 3: select studies

EndNote 8X software was used to manage retrieved references. Duplicate articles were removed based on the four-step SRA-DM algorithm and manually removed in the final step (Levac et al., 2010).

The screening was initially conducted by titles (NN and HT) and then abstracted by two of the authors separately (NN, SGH). To confirm the agreement between the evaluators, 10% (*n* = 1,154) of the articles were evaluated. In the next step, the full text of the articles was evaluated by two independent reviewers (NN, SGH). Any disagreements or disputes between the two evaluators were settled by agreement, and then by the third evaluator (JSH). This review was registered (HT) on the Open Science Framework (10.17605/OSF.IO/3MWGJ).

#### 2.3.1. Methodological quality appraisal

We did not appraise the methodological quality or risk of bias of the included articles, being consistent with guidance on conducting scoping reviews (Peters et al., 2015).

### 2.4. Stage 4: chart the data

The data elements we included were chosen through negotiation among authors (HT, MF, JSH, and AB, SE), and all extracted data were frequently reviewed among team members. The data extracted in this study included the first author/year, country, study participants, sample size, aims of the study, recruitment setting, study design, method of data collection, outcome (measures), IBD patient type, disease duration, kind of identified needs, and key findings as reported in the paper.

Two authors (NN, SGH) separately performed diagram form design and information extraction; any disagreement and discordance were resolved at first through discussion and then by the third evaluator (AB).

#### 2.4.1. Operational definition of domains of need

Based on the current literature and clinical expertise, individual supportive care needs are divided into eleven areas, including psychological/emotional, physical, spiritual, informational, family-related needs, social, interpersonal intimacy needs, practical, daily living needs, patient-clinician communication, and support needs related to the future (Fitch, 2008; Ream et al., 2008; Butow et al., 2012; Carey et al., 2012; Ford et al., 2012; Cockle-Hearne et al., 2013). The classification of supportive care needs was presented using the Supportive Care Needs Framework (Fitch, 2008) and the current definition of “supportive care needs” (Hui, 2014).

### 2.5. Stage 5: collate, summarize, analyze, and report the results

All quantitative and descriptive data of the studies included in this scoping review were collected. Additionally, a thematic synthesis based on the adductive technique was used for all data (Peters et al., 2015). Researchers generated and extracted themes, sub-themes, and key factors by using a theoretical framework, discussing major and minor themes, and examining examples through sharing and discussing key themes, subthemes, and exemplar quotes with the team members in articles (NN, HT, MF, JSH, AB, SE, and SGH). Two independent reviewers (N.N. and M.F.) completed this process, and resolved any differences through discussion.

### 2.6. Stage 6: consultation with stakeholders

The purpose of this step was to measure stakeholders' insights into the findings of this review through consultation with them and to increase its methodological accuracy (Martin et al., 1992; Arksey and O'Malley, 2005). Information, information resources, and supportive needs of adults over 18 with IBD were assessed. In addition, a group of researchers and healthcare professionals specializing in IBD was consulted to explore their perspectives and search for more relevant articles. The main research questions include information, support, and psychological needs. The first author (NN) approached professional stakeholders by telephone and email. Expert groups were invited to complete an online survey (duration: ~15 min) that included open-ended and closed-ended questions in Avalform©, a safe, electronic data gathering and management platform. Themes and explanations of our review's findings were finalized after the council and consultation with stakeholders.

## 3. Results

### 3.1. Study selection and characteristics

**Figure 1:**
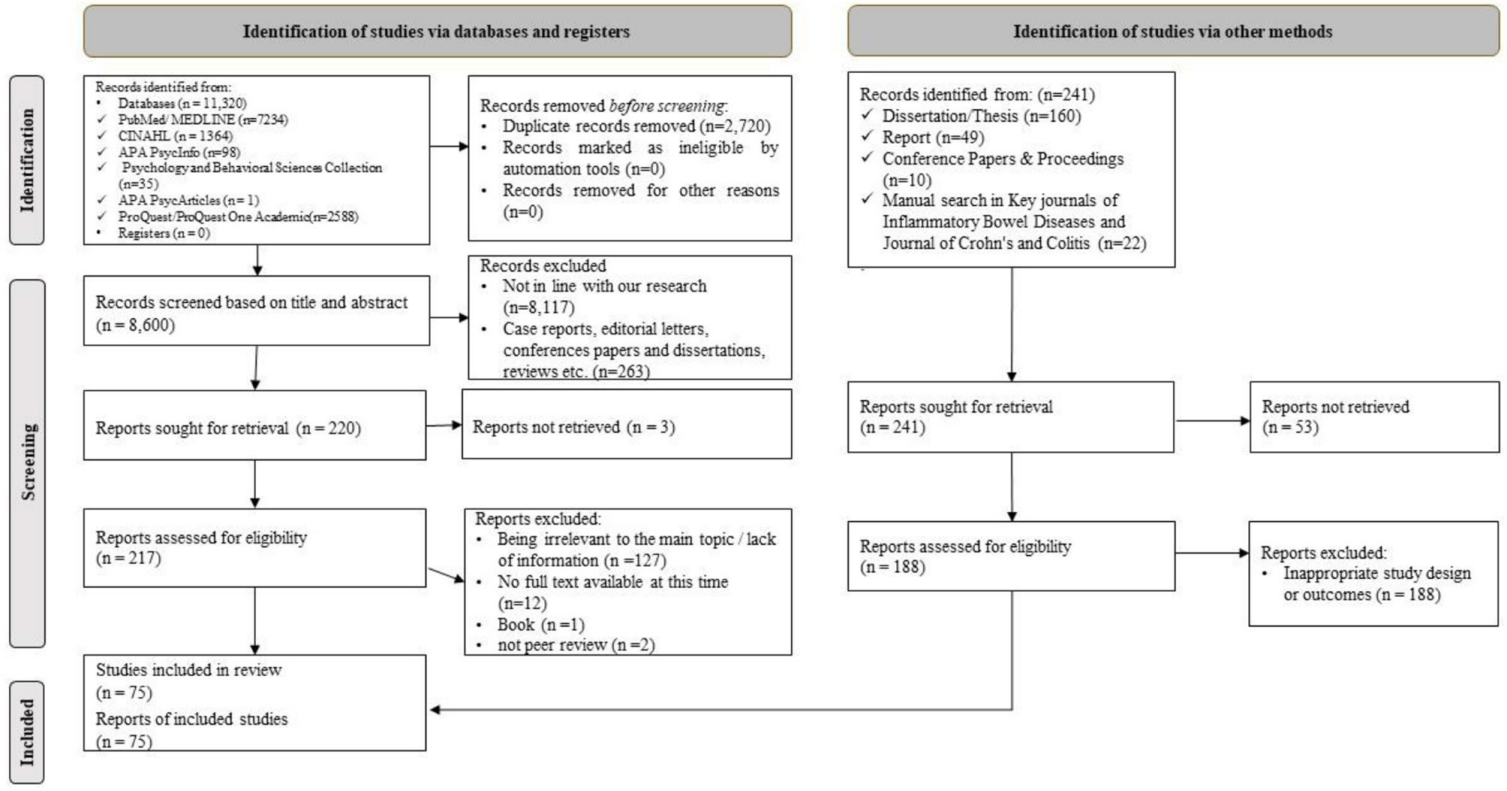
Flow diagram of the study selection process.

According to Figure 1, 11,539 articles were found. Of the studies, 8,602 remained after the removal of duplicate articles. After reviewing these articles, 217 articles were selected to review the full text. Then, 140 articles were removed from the study due to non-compliance with the subject of the study, lack of full text, books, and no peer review. Finally, 75 studies (O'Sullivan et al., 2000; Quan et al., 2003; Ryan et al., 2003; Rezailashkajani et al., 2006; Politi et al., 2008; Cullen et al., 2010; Molnár et al., 2010; Subasinghe et al., 2010; Bernstein et al., 2011; Conrad et al., 2012; Wong et al., 2012; Yeung et al., 2012; Huang et al., 2013; Mukewar et al., 2013; Selinger et al., 2013; Viazis et al., 2013; Greveson, 2014; Lahat et al., 2014; Lesnovska et al., 2014, 2017; Pham et al., 2014; Shepherd et al., 2014; Becker et al., 2015; Burkhalter et al., 2015; Catalán-Serra et al., 2015; Yoo et al., 2015; Berding et al., 2016; Bray et al., 2016; Greveson et al., 2016; Khan et al., 2016; Pittet et al., 2016; Schoultz, 2016; Schoultz et al., 2016; Sephton et al., 2016; Wheat et al., 2016; Britt, 2017; Larsson et al., 2017; López-Sanromán et al., 2017; Niv et al., 2017; Restall et al., 2017; Wilburn et al., 2017; Cho et al., 2018; Jordan et al., 2018; Kamp and Brittain, 2018; Knowles et al., 2018; Martin-Fernandez et al., 2018; McDermott et al., 2018; Philip et al., 2018; Wu and Zhong, 2018; Daher et al., 2019; Sarwan et al., 2019; Wåhlin et al., 2019; Yu et al., 2019; Casellas et al., 2020; Del Hoyo et al., 2020; Feng et al., 2020; Karadag et al., 2020; Keller et al., 2020; Khalil et al., 2020; Moon et al., 2020; Santos et al., 2020; Zare et al., 2020; Al Khoury et al., 2021; Aluzaite et al., 2021; Cai et al., 2021; Chan et al., 2021; Fawson et al., 2021; Goodsall et al., 2021; Graffigna et al., 2021; Kutschera et al., 2021; Long et al., 2021; Popov et al., 2021; Rubin et al., 2021; Volpato et al., 2021; Vutcovici et al., 2021; Goren et al., 2022) remained in this scoping review. There were 74 articles in English and one study in German. The design of the articles was cross-sectional (*n* = 27, 36.00%), qualitative (*n* = 24, 32.00%), mixed-method (*n* = 7, 9.33%), review (*n* = 6, 8.00%), cohort (*n* = 4, 5.33%), interventional (*n* = 2, 2.67%), and others (*n* = 5, 6.67%). Out of 75 studies, 62 and 53 studies were concerned with the examination of information needs and supportive needs, respectively (Supplementary Table 3).

### 3.2. Thematic synthesis

Informational, information resources and supportive needs of adults with IBD are presented under main thematic and sub-thematic headings.

### 3.3. Informational needs of patients with IBD

Nearly 55 items of informational needs were identified and listed in Table 1. Three most pronounced needs in this section were “nutrition/diet information” following by “medications and side effects information”, and “cure/treatment and side effects”, which constituted the subject of interest in 49.33, 38.67, and 32.00% of the retrieved papers, respectively. It is obvious that every of these items possesses unique importance. Diet, if not the leading, but is one of the critical environmental determinants in the course of IBD that greatly affects self-healing and regeneration capacity of the gut (Skrautvol, 2011). It was revealed that patients with IBD are often concerned and fearful about the side effects of medications that may alter their future living (Thompson et al., 2016). Chronic and heterogeneous nature of IBD necessitate the treatment target to be long-lasting remission in order to prevent possible disease complications and progression. Therapeutic failure may result from differences in drug responses in terms of efficacy and toxicity due to variability between individuals (Voskuil et al., 2019), and consequently this issue has been identified as a vital need for such patients. Other important informational needs that were identified include “extra-intestinal manifestations/disease complications (29.33%)”, “symptoms/clinical manifestations (28.00%)”, “surgery information with (28.00%)”, “gynecological issues (28.00%)”, “etiology/cause of disease (26.67%)”, “new research and advances (22.67%)”, and “prognosis/long-term consequences (20.00%)”.

**Table 1:** Informational needs.

**Information needs subcategory (*n*, %)**	**References**
General information about IBD (*n* = 16, 21.33%)	(Molnár et al., 2010; Subasinghe et al., 2010; Conrad et al., 2012; Mukewar et al., 2013; Yoo et al., 2015; Khan et al., 2016; Pittet et al., 2016; Sephton et al., 2016; Wheat et al., 2016; Larsson et al., 2017; Niv et al., 2017; Cho et al., 2018; Wu and Zhong, 2018; Yu et al., 2019; Del Hoyo et al., 2020; Graffigna et al., 2021)
Etiology/Cause of IBD information (*n* = 20, 26.67%)	(O'Sullivan et al., 2000; Rezailashkajani et al., 2006; Molnár et al., 2010; Bernstein et al., 2011; Conrad et al., 2012; Wong et al., 2012; Mukewar et al., 2013; Viazis et al., 2013; Lesnovska et al., 2014; Becker et al., 2015; Pittet et al., 2016; Wheat et al., 2016; López-Sanromán et al., 2017; Restall et al., 2017; Martin-Fernandez et al., 2018; McDermott et al., 2018; Wu and Zhong, 2018; Daher et al., 2019; Casellas et al., 2020; Karadag et al., 2020)
Epidemiological and pathogenesis results in information (*n* = 4, 5.33%)	(Rezailashkajani et al., 2006; Berding et al., 2016; Pittet et al., 2016; Wheat et al., 2016)
IBD evolution and further course information (*n* = 8, 10.67%)	(Conrad et al., 2012; Mukewar et al., 2013; Lesnovska et al., 2014; Becker et al., 2015; Pittet et al., 2016; López-Sanromán et al., 2017; Martin-Fernandez et al., 2018; Casellas et al., 2020)
Symptoms/ clinical manifestations of IBD information (*n* = 21, 28.00%)	(Rezailashkajani et al., 2006; Molnár et al., 2010; Bernstein et al., 2011; Wong et al., 2012; Mukewar et al., 2013; Lahat et al., 2014; Lesnovska et al., 2014, 2017; Becker et al., 2015; Catalán-Serra et al., 2015; Kamp and Brittain, 2018; Martin-Fernandez et al., 2018; McDermott et al., 2018; Wu and Zhong, 2018; Daher et al., 2019; Casellas et al., 2020; Karadag et al., 2020; Keller et al., 2020; Zare et al., 2020; Al Khoury et al., 2021; Volpato et al., 2021)
Anatomy/physiology of gastrointestinal system information (*n* = 4, 5.33%)	(Rezailashkajani et al., 2006; Lesnovska et al., 2014; Berding et al., 2016; Volpato et al., 2021)
Diagnosis information (*n* = 8, 10.67%)	(Rezailashkajani et al., 2006; Molnár et al., 2010; Mukewar et al., 2013; Lesnovska et al., 2014; Yoo et al., 2015; Berding et al., 2016; Casellas et al., 2020; Volpato et al., 2021)
Diagnostic methods, risk, and interpretation information (*n* = 4, 5.33%)	(O'Sullivan et al., 2000; Catalán-Serra et al., 2015; Martin-Fernandez et al., 2018; Goren et al., 2022)
Prognosis/ long-term consequences information (*n* = 15, 20.00%)	(O'Sullivan et al., 2000; Quan et al., 2003; Bernstein et al., 2011; Wong et al., 2012; Becker et al., 2015; Wheat et al., 2016; Restall et al., 2017; Kamp and Brittain, 2018; McDermott et al., 2018; Wu and Zhong, 2018; Daher et al., 2019; Yu et al., 2019; Casellas et al., 2020; Al Khoury et al., 2021; Volpato et al., 2021)
Risk factors of flares information (*n* = 4, 5.33%)	(Quan et al., 2003; Pittet et al., 2016; Yu et al., 2019; Del Hoyo et al., 2020)
The prevention of relapse/ disease control information (*n* = 8, 10.67%)	(O'Sullivan et al., 2000; Lesnovska et al., 2014; Khan et al., 2016; Wheat et al., 2016; Britt, 2017; Kamp and Brittain, 2018; Wu and Zhong, 2018; Zare et al., 2020)
Action in relapse/ treatment of current relapse information (*n* = 2, 2.67%)	(Molnár et al., 2010; Lesnovska et al., 2017)
Extra-intestinal manifestations / IBD complications information (*n* = 22, 29.33%)	(O'Sullivan et al., 2000; Quan et al., 2003; Rezailashkajani et al., 2006; Politi et al., 2008; Molnár et al., 2010; Subasinghe et al., 2010; Bernstein et al., 2011; Wong et al., 2012; Huang et al., 2013; Mukewar et al., 2013; Viazis et al., 2013; Lesnovska et al., 2014; Becker et al., 2015; Berding et al., 2016; Pittet et al., 2016; Britt, 2017; Niv et al., 2017; Martin-Fernandez et al., 2018; Wu and Zhong, 2018; Daher et al., 2019; Casellas et al., 2020; Al Khoury et al., 2021)
Cancer information (*n* = 14, 18.67%)	(O'Sullivan et al., 2000; Quan et al., 2003; Molnár et al., 2010; Bernstein et al., 2011; Wong et al., 2012; Viazis et al., 2013; Catalán-Serra et al., 2015; Pittet et al., 2016; Sephton et al., 2016; Wu and Zhong, 2018; Daher et al., 2019; Casellas et al., 2020; Al Khoury et al., 2021; Volpato et al., 2021)
Risk of infection information (*n* = 1, 1.33%)	(Molnár et al., 2010)
Comorbidity and its management information (*n* = 2, 2.67%)	(Restall et al., 2017; Casellas et al., 2020)
Mortality information (*n* = 4, 5.33%)	(Catalán-Serra et al., 2015; Pittet et al., 2016; Al Khoury et al., 2021; Volpato et al., 2021)
Cure/treatment and side effects information (*n* = 24, 32.00%)	(O'Sullivan et al., 2000; Quan et al., 2003; Politi et al., 2008; Molnár et al., 2010; Conrad et al., 2012; Wong et al., 2012; Becker et al., 2015; Catalán-Serra et al., 2015; Berding et al., 2016; Pittet et al., 2016; Britt, 2017; Lesnovska et al., 2017; López-Sanromán et al., 2017; Restall et al., 2017; Cho et al., 2018; Martin-Fernandez et al., 2018; Wu and Zhong, 2018; Casellas et al., 2020; Karadag et al., 2020; Keller et al., 2020; Khalil et al., 2020; Zare et al., 2020; Al Khoury et al., 2021; Volpato et al., 2021)
Medications and side effects information (*n* = 29, 38.67%)	(O'Sullivan et al., 2000; Rezailashkajani et al., 2006; Cullen et al., 2010; Molnár et al., 2010; Subasinghe et al., 2010; Bernstein et al., 2011; Conrad et al., 2012; Wong et al., 2012; Mukewar et al., 2013; Viazis et al., 2013; Lesnovska et al., 2014; Becker et al., 2015; Yoo et al., 2015; Pittet et al., 2016; Sephton et al., 2016; Wheat et al., 2016; Niv et al., 2017; Cho et al., 2018; Kamp and Brittain, 2018; McDermott et al., 2018; Wu and Zhong, 2018; Daher et al., 2019; Yu et al., 2019; Casellas et al., 2020; Del Hoyo et al., 2020; Khalil et al., 2020; Santos et al., 2020; Zare et al., 2020; Volpato et al., 2021)
New research and advances in IBD information (*n* = 17, 22.67%)	(O'Sullivan et al., 2000; Politi et al., 2008; Conrad et al., 2012; Wong et al., 2012; Mukewar et al., 2013; Catalán-Serra et al., 2015; Pittet et al., 2016; Sephton et al., 2016; Wheat et al., 2016; Lesnovska et al., 2017; Niv et al., 2017; Kamp and Brittain, 2018; Yu et al., 2019; Casellas et al., 2020; Khalil et al., 2020; Al Khoury et al., 2021; Volpato et al., 2021)
Participate in research information (*n* = 1, 1.33%)	(Casellas et al., 2020)
Importance of adherence to medication information (*n* = 2, 2.67%)	(Wheat et al., 2016; Casellas et al., 2020)
Surgery information (*n* = 21, 28.00%)	(O'Sullivan et al., 2000; Quan et al., 2003; Rezailashkajani et al., 2006; Molnár et al., 2010; Bernstein et al., 2011; Conrad et al., 2012; Wong et al., 2012; Mukewar et al., 2013; Viazis et al., 2013; Lesnovska et al., 2014; Catalán-Serra et al., 2015; Pittet et al., 2016; Niv et al., 2017; Restall et al., 2017; Martin-Fernandez et al., 2018; McDermott et al., 2018; Wu and Zhong, 2018; Daher et al., 2019; Yu et al., 2019; Casellas et al., 2020; Volpato et al., 2021)
No medication therapies information (*n* = 3, 4.00%)	(O'Sullivan et al., 2000; Rezailashkajani et al., 2006; Casellas et al., 2020)
Alternative and complementary medicines information (*n* = 14, 18.67%)	(Molnár et al., 2010; Conrad et al., 2012; Mukewar et al., 2013; Becker et al., 2015; Pittet et al., 2016; Sephton et al., 2016; Wheat et al., 2016; Niv et al., 2017; Cho et al., 2018; Wu and Zhong, 2018; Daher et al., 2019; Casellas et al., 2020; Khalil et al., 2020; Goren et al., 2022)
COVID-19 information (*n* = 3, 4.00%)	(Karadag et al., 2020; Goodsall et al., 2021; Long et al., 2021)
Vaccination information (*n* = 8, 10.67%)	(Molnár et al., 2010; Yeung et al., 2012; Catalán-Serra et al., 2015; Pittet et al., 2016; Casellas et al., 2020; Aluzaite et al., 2021; Chan et al., 2021; Goren et al., 2022)
Lifestyle and daily life information (*n* = 10, 13.33%)	(Lesnovska et al., 2014; Yoo et al., 2015; Restall et al., 2017; McDermott et al., 2018; Wu and Zhong, 2018; Yu et al., 2019; Casellas et al., 2020; Khalil et al., 2020; Al Khoury et al., 2021; Goren et al., 2022)
Risky behaviors information (*n* = 6, 8.00%)	(Quan et al., 2003; Ryan et al., 2003; Cho et al., 2018; Martin-Fernandez et al., 2018; Cai et al., 2021; Goren et al., 2022)
Nutrition/ Diet information (*n* = 37, 49.33%)	(O'Sullivan et al., 2000; Rezailashkajani et al., 2006; Molnár et al., 2010; Subasinghe et al., 2010; Bernstein et al., 2011; Conrad et al., 2012; Wong et al., 2012; Mukewar et al., 2013; Viazis et al., 2013; Lesnovska et al., 2014, 2017; Becker et al., 2015; Catalán-Serra et al., 2015; Yoo et al., 2015; Berding et al., 2016; Pittet et al., 2016; Sephton et al., 2016; Wheat et al., 2016; Larsson et al., 2017; Niv et al., 2017; Restall et al., 2017; Cho et al., 2018; Kamp and Brittain, 2018; Martin-Fernandez et al., 2018; McDermott et al., 2018; Wu and Zhong, 2018; Daher et al., 2019; Yu et al., 2019; Casellas et al., 2020; Del Hoyo et al., 2020; Keller et al., 2020; Khalil et al., 2020; Al Khoury et al., 2021; Cai et al., 2021; Fawson et al., 2021; Volpato et al., 2021; Goren et al., 2022)
Nutritional deficiencies information (*n* = 3, 4.00%)	(Daher et al., 2019; Casellas et al., 2020; Cai et al., 2021)
Nutritional supplement information (*n* = 7, 9.33%)	(Bernstein et al., 2011; Wong et al., 2012; Wu and Zhong, 2018; Daher et al., 2019; Casellas et al., 2020; Cai et al., 2021; Goren et al., 2022)
Exercise/physical activity information (*n* = 6, 8.00%)	(Becker et al., 2015; Catalán-Serra et al., 2015; Cho et al., 2018; Casellas et al., 2020; Khalil et al., 2020; Fawson et al., 2021)
Rehabilitation information (*n* = 1, 1.33%)	(Conrad et al., 2012)
IBD-related travel information (*n* = 7, 9.33%)	(Greveson, 2014; Shepherd et al., 2014; Catalán-Serra et al., 2015; Greveson et al., 2016; Philip et al., 2018; Casellas et al., 2020; Aluzaite et al., 2021)
Pain and symptom management information (*n* = 5, 6.67%)	(Bernstein et al., 2011; Wong et al., 2012; Britt, 2017; Kamp and Brittain, 2018; Daher et al., 2019)
Disease management information (*n* = 17, 22.67%)	(Bernstein et al., 2011; Conrad et al., 2012; Wong et al., 2012; Lesnovska et al., 2014, 2017; Berding et al., 2016; Pittet et al., 2016; Sephton et al., 2016; Wheat et al., 2016; Britt, 2017; Larsson et al., 2017; Martin-Fernandez et al., 2018; Wu and Zhong, 2018; Daher et al., 2019; Casellas et al., 2020; Del Hoyo et al., 2020; Al Khoury et al., 2021)
Tip for coping information (*n* = 10, 13.33%)	(Bernstein et al., 2011; Wong et al., 2012; Berding et al., 2016; Sephton et al., 2016; Lesnovska et al., 2017; McDermott et al., 2018; Wu and Zhong, 2018; Daher et al., 2019; Khalil et al., 2020; Volpato et al., 2021)
Communication aspects information (*n* = 9, 12.00%)	(Politi et al., 2008; Becker et al., 2015; Pittet et al., 2016; Larsson et al., 2017; Niv et al., 2017; Cho et al., 2018; Yu et al., 2019; Casellas et al., 2020; Karadag et al., 2020)
Stories and experiences about defeating the disease information (*n* = 2, 2.67%)	(Yu et al., 2019; Khalil et al., 2020)
Psychological factors/dealing with stress (*n* = 12, 16.00%)	(O'Sullivan et al., 2000; Mukewar et al., 2013; Becker et al., 2015; Pittet et al., 2016; Restall et al., 2017; Daher et al., 2019; Casellas et al., 2020; Del Hoyo et al., 2020; Khalil et al., 2020; Cai et al., 2021; Fawson et al., 2021; Goren et al., 2022)
Quality of life information (*n* = 4, 5.33%)	(Mukewar et al., 2013; Khan et al., 2016; Pittet et al., 2016; Lesnovska et al., 2017)
Religious topics information (*n* = 1, 1.33%)	(Daher et al., 2019)
Gynecological issues information (*n* = 21, 28.00%)	(O'Sullivan et al., 2000; Quan et al., 2003; Rezailashkajani et al., 2006; Molnár et al., 2010; Bernstein et al., 2011; Wong et al., 2012; Selinger et al., 2013; Catalán-Serra et al., 2015; Yoo et al., 2015; Berding et al., 2016; Pittet et al., 2016; Sephton et al., 2016; Lesnovska et al., 2017; Cho et al., 2018; Martin-Fernandez et al., 2018; Wu and Zhong, 2018; Daher et al., 2019; Casellas et al., 2020; Al Khoury et al., 2021; Volpato et al., 2021; Goren et al., 2022)
Sexuality information (*n* = 2, 2.67%)	(Becker et al., 2015; Casellas et al., 2020)
Heredity/ genetic/microbiome information (*n* = 14, 18.67%)	(O'Sullivan et al., 2000; Politi et al., 2008; Molnár et al., 2010; Bernstein et al., 2011; Wong et al., 2012; Selinger et al., 2013; Becker et al., 2015; Catalán-Serra et al., 2015; Pittet et al., 2016; Lesnovska et al., 2017; Daher et al., 2019; Casellas et al., 2020; Volpato et al., 2021; Goren et al., 2022)
Family/significant others informing information (*n* = 9, 12.00%)	(Bernstein et al., 2011; Wong et al., 2012; Lahat et al., 2014; Lesnovska et al., 2014; Restall et al., 2017; Wu and Zhong, 2018; Daher et al., 2019; Khalil et al., 2020; Cai et al., 2021)
Family-related information (*n* = 3, 4.00%)	(Viazis et al., 2013; Restall et al., 2017; Martin-Fernandez et al., 2018)
Work-related issues information (*n* = 9, 12.00%)	(Bernstein et al., 2011; Conrad et al., 2012; Wong et al., 2012; Viazis et al., 2013; Becker et al., 2015; Catalán-Serra et al., 2015; Restall et al., 2017; Daher et al., 2019; Casellas et al., 2020)
Health care measures regarding IBD/ preventive care information (*n* = 1, 1.33%)	(Conrad et al., 2012)
Accessing resources to obtain medical care and socio-sanitary resources information (*n* = 2, 2.67%)	(Wheat et al., 2016; Casellas et al., 2020)
Hospitals and doctors' information (*n* = 4, 5.33%)	(Conrad et al., 2012; Wheat et al., 2016; Lesnovska et al., 2017; Niv et al., 2017)
When connecting to the IBD team/ specialist referrals information (*n* = 6, 8.00%)	(Bernstein et al., 2011; Wong et al., 2012; Daher et al., 2019; Casellas et al., 2020; Al Khoury et al., 2021; Goren et al., 2022)
Legal and policy aspects of information (*n* = 9, 12.00%)	(Conrad et al., 2012; Lahat et al., 2014; Catalán-Serra et al., 2015; Pittet et al., 2016; Daher et al., 2019; Yu et al., 2019; Casellas et al., 2020; Al Khoury et al., 2021; Volpato et al., 2021)
Insurance/ finance information (*n* = 7, 9.33%)	(Bernstein et al., 2011; Conrad et al., 2012; Wong et al., 2012; Restall et al., 2017; Cho et al., 2018; Daher et al., 2019; Khalil et al., 2020)

### 3.4. Information sources and methods of patients with IBD

Five categories including “health care providers (44.00%)”, “media (38.67%)”, “interpersonal communications (28.00%)”, “printed materials (25.33%)”, and “scientific sources (9.33%)” were totally identified in this section (Table 2). Managing IBD, an emerging global disease needs a group of health care professionals possibly for lifelong (Prasad et al., 2020). This disease is a burdensome one, and quality of life of patients with IBD is improved *via* optimal quality of care, which is practical through a multidisciplinary team approach (Ricci et al., 2008; Panés et al., 2014; Sandborn et al., 2014). Despite the availability of a huge amount of different information sources, the favorite ones for these patients are still gastroenterologists and medical professionals (Bernstein et al., 2011; Wong et al., 2012; Huang et al., 2013). However, communication and collaborative technologies have reshaped the interactions of patients with healthcare providers. Different types of media, for instance social media, form a platform for patients to instantly discuss issues, find out new reports, analyze investigations, communicate with peers, gain information from crowd-sources, looking for support, and give advice to others (McCracken, 2012). Indeed, health centers are increasingly using such ways (Hawker, 2010; Van de Belt et al., 2012) to provide a better experience of care at a more cost-efficient manner (Van de Belt et al., 2013). It should be noted that course of the disease determines consulted sources of information. It is important for the patients to gain information in a progressive and sparing manner in concordance with the disease course and treatments. To substantiate, it was revealed that there should be a balance between information given by gastroenterologists and actual experience of the patient from the disease (Pittet et al., 2016). Social and family life of the patients with IBD become impaired (Zutshi et al., 2007; Viazis et al., 2013), and this imposes a negative impact on their quality of life (Casellas et al., 1999). Intriguingly, those with high quality of life significantly search less information (Pittet et al., 2016).

**Table 2:** Information sources and methods.

**Category (%)**	**Subcategory**	**References**
Health provider team (*n* = 33, 44.00%)	Health professionals' team Medical specialists/ physicians/ gastroenterologists Family doctor general practitioner/ Primary care physician Nurse Dieticians Physiotherapists Psychiatrists/psychologist Pharmacist Surgeon Naturopaths/ complementary medicine Hospitals/ IBD clinic Public health department	(Cullen et al., 2010; Molnár et al., 2010; Bernstein et al., 2011; Conrad et al., 2012; Wong et al., 2012; Yeung et al., 2012; Huang et al., 2013; Mukewar et al., 2013; Viazis et al., 2013; Greveson, 2014; Shepherd et al., 2014; Becker et al., 2015; Catalán-Serra et al., 2015; Berding et al., 2016; Greveson et al., 2016; Pittet et al., 2016; López-Sanromán et al., 2017; Restall et al., 2017; Cho et al., 2018; Martin-Fernandez et al., 2018; McDermott et al., 2018; Philip et al., 2018; Wu and Zhong, 2018; Daher et al., 2019; Yu et al., 2019; Casellas et al., 2020; Karadag et al., 2020; Al Khoury et al., 2021; Aluzaite et al., 2021; Chan et al., 2021; Long et al., 2021; Volpato et al., 2021; Vutcovici et al., 2021)
Printed materials (*n* = 19, 25.33%)	Books Brochures/ booklet/ leaflet/ pamphlets Magazines/ newspapers/ press Materials from physician	(Politi et al., 2008; Bernstein et al., 2011; Conrad et al., 2012; Wong et al., 2012; Huang et al., 2013; Mukewar et al., 2013; Greveson, 2014; Shepherd et al., 2014; Becker et al., 2015; Catalán-Serra et al., 2015; Greveson et al., 2016; Pittet et al., 2016; Restall et al., 2017; McDermott et al., 2018; Philip et al., 2018; Wu and Zhong, 2018; Karadag et al., 2020; Long et al., 2021; Volpato et al., 2021)
Media (*n* = 29, 38.67%)	TV/ radio/ or videos Internet searching Website Social media Applications Online sources Telephone information service E-mail	(Politi et al., 2008; Cullen et al., 2010; Molnár et al., 2010; Bernstein et al., 2011; Conrad et al., 2012; Wong et al., 2012; Huang et al., 2013; Viazis et al., 2013; Greveson, 2014; Shepherd et al., 2014; Becker et al., 2015; Catalán-Serra et al., 2015; Berding et al., 2016; Greveson et al., 2016; Pittet et al., 2016; Niv et al., 2017; Restall et al., 2017; Cho et al., 2018; Kamp and Brittain, 2018; Martin-Fernandez et al., 2018; McDermott et al., 2018; Philip et al., 2018; Wu and Zhong, 2018; Yu et al., 2019; Karadag et al., 2020; Al Khoury et al., 2021; Long et al., 2021; Volpato et al., 2021; Vutcovici et al., 2021)
Interpersonal communication (*n* = 21, 28.00%)	Friends/ family/ acquaintances Personal experience/ other patients Health insurance Counseling, support groups/ support services/ patients' association meetings Travel Clinic Legal representation Pharmaceutical companies/ research institutions	(Politi et al., 2008; Cullen et al., 2010; Bernstein et al., 2011; Conrad et al., 2012; Wong et al., 2012; Huang et al., 2013; Mukewar et al., 2013; Greveson, 2014; Shepherd et al., 2014; Becker et al., 2015; Catalán-Serra et al., 2015; Greveson et al., 2016; Pittet et al., 2016; Restall et al., 2017; Cho et al., 2018; Martin-Fernandez et al., 2018; McDermott et al., 2018; Philip et al., 2018; Wu and Zhong, 2018; Yu et al., 2019; Long et al., 2021)
Scientific sources (*n* = 7, 9.33%)	Scientific and medical journal articles Conferences paper Books Encyclopedia Research summaries with IBD Doctor-mediated guidance	(Conrad et al., 2012; Yeung et al., 2012; Huang et al., 2013; Catalán-Serra et al., 2015; Restall et al., 2017; Wu and Zhong, 2018; Yu et al., 2019)

### 3.5. Supportive needs of patients with IBD

In this section, “patient education needs” was mentioned in 49.33% of the retrieved papers and is the leading subject in supportive needs category. Next ranks are allocated to “psychological/mental health support (29.33%)”, “social health support systems and support groups (20.00%)”, and “health care access (18.67%)”. However, there are a variety of other supportive needs that are listed in Table 3 such as physical needs, spiritual needs, and family-related needs. During the recent years, the traditional central role of physicians in clinical decision-making has changed toward patient-centered care (Morioka, 1988). However, participation of patients in expressing informed preferences and self-management practices is restricted due to lack of sufficient information (Coulter et al., 1999). The fundamental value of patient contribution is widely accepted. According to the guidelines of European Crohns and Colitis Organization, quality of care in IBD will be optimized if patients are provided with sufficient and appropriate information and education (Elkjaer et al., 2008). However, there are enormous heterogeneity in education practices throughout the regions (Burisch et al., 2014). Due to the importance of “psychological/emotional needs”, this item is specifically discussed in the following.

**Table 3:** Supportive needs.

	**Support needs subcategory (*n*, %)**	**Details of subcategory's needs**	**References**
Psychological/ Emotional needs	Psychological/ Emotional disaster needs (*n* = 8, 10.67%)	Feeling normal, Body image, Talk to patients without caregivers, impact on mental or emotional health, feel comfortable talking to their physician about emotional concerns, Attractiveness, Continence, Clear mindedness, Role, Pleasure, Security, and Continence, reduce anxiety, empathizing with fellow group members/caregivers of Crohn's and UC, understanding the challenges associated with Crohn's and UC, psychosocial concerns,	(Bray et al., 2016; Britt, 2017; Wilburn et al., 2017; Cho et al., 2018; Kamp and Brittain, 2018; Long et al., 2021; Rubin et al., 2021; Vutcovici et al., 2021)
	Psychological screening and assessment (*n* = 6, 8.00%)	assess psychological symptoms and patients' ability to cope with illness, for each patient stressors and Emotional distress support, Ask patients about their concerns in the psychosocial domain and emotional health, Psychological Screening and support; identify and orient patients with mental health difficulties to a mental health service by the gastroenterology team,	(O'Sullivan et al., 2000; Burkhalter et al., 2015; Pittet et al., 2016; López-Sanromán et al., 2017; Cho et al., 2018; Volpato et al., 2021)
	Psychological /mental health support (*n* = 22, 29.33%)	shorten the follow-up intervals when a screening is positive, psychological interventions (Psychoanalytic, psychodynamic, and cognitive-behavioral therapy (CBT, pharmacology intervention); Emotional support from a health care professionals, Access to psychological counseling, emotional support form of family and friends, integrated psychosomatic support and psychotherapy (Psychodynamic therapy / Humanistic therapy), Mindfulness, meditation, yoga, and Pilates exercises, Distracting oneself, To periodically address the ‘fears and limitations of living with UC, manage the worry, improvement in the quality of life (During Remission),	(O'Sullivan et al., 2000; Burkhalter et al., 2015; Berding et al., 2016; Khan et al., 2016; Schoultz, 2016; Schoultz et al., 2016; Larsson et al., 2017; Cho et al., 2018; Jordan et al., 2018; Knowles et al., 2018; Casellas et al., 2020; Karadag et al., 2020; Zare et al., 2020; Al Khoury et al., 2021; Fawson et al., 2021; Graffigna et al., 2021; Kutschera et al., 2021; Long et al., 2021; Popov et al., 2021; Rubin et al., 2021; Volpato et al., 2021; Vutcovici et al., 2021)
	Coping with disease (*n* = 8, 10.67%)	ability to cope with the disease, all the side effects, negative emotions, Disease acceptance,	(Viazis et al., 2013; Berding et al., 2016; Schoultz, 2016; Schoultz et al., 2016; Larsson et al., 2017; Zare et al., 2020; Fawson et al., 2021; Vutcovici et al., 2021)
	Psychological self-care (*n* = 4, 5.33%)	ability to Stress control, Self-esteem, Self-efficacy,	(Berding et al., 2016; Wilburn et al., 2017; Sarwan et al., 2019; Zare et al., 2020)
Physical needs	Physical symptoms (*n* = 5, 6.67%)	tiredness, fatigue, and pain management, coping and handling symptoms,	(Schoultz et al., 2016; Jordan et al., 2018; Kamp and Brittain, 2018; Aluzaite et al., 2021; Vutcovici et al., 2021)
Spiritual needs	Practice religious beliefs, and existential concerns (*n* = 2, 2.67%)	Positive attitude to life	(Cho et al., 2018; Zare et al., 2020)
Informational needs	Ability to obtain information (*n* = 1, 1.33%)	Ability to obtain the source of information,	(Zare et al., 2020)
	Patient education (*n* = 37, 49.33%)	sufficient information, informed treatment decisions,	(Quan et al., 2003; Ryan et al., 2003; Politi et al., 2008; Cullen et al., 2010; Yeung et al., 2012; Selinger et al., 2013; Viazis et al., 2013; Lesnovska et al., 2014, 2017; Berding et al., 2016; Bray et al., 2016; Pittet et al., 2016; Schoultz, 2016; Schoultz et al., 2016; Sephton et al., 2016; Britt, 2017; Larsson et al., 2017; López-Sanromán et al., 2017; Restall et al., 2017; Cho et al., 2018; Kamp and Brittain, 2018; Martin-Fernandez et al., 2018; Philip et al., 2018; Casellas et al., 2020; Khalil et al., 2020; Santos et al., 2020; Zare et al., 2020; Al Khoury et al., 2021; Cai et al., 2021; Chan et al., 2021; Fawson et al., 2021; Goodsall et al., 2021; Graffigna et al., 2021; Popov et al., 2021; Rubin et al., 2021; Volpato et al., 2021; Vutcovici et al., 2021)
	Sources of Information (*n* = 2, 2.67%)	IBD knowledge	(Khan et al., 2016; Philip et al., 2018)
	Provide clear information (*n* = 6, 8.00%)	Information at the right time, clear, structured, and factual	(Pittet et al., 2016; Schoultz et al., 2016; Lesnovska et al., 2017; Karadag et al., 2020; Al Khoury et al., 2021; Goodsall et al., 2021)
Family-related needs	Family, friends, and colleagues' education/ informing (*n* = 8, 10.67%)	increasing awareness about general characteristics of the disease and improving educational resources for families, and the general public, family informing, patients wanted their partner to receive more medical information from the physician about their physical condition,	(Bernstein et al., 2011; Wong et al., 2012; Lahat et al., 2014; Lesnovska et al., 2014; Restall et al., 2017; Karadag et al., 2020; Cai et al., 2021; Popov et al., 2021)
	Attention to family/caregivers of the patient (*n* = 5, 6.67%)	emotional and practical support of Family and partners,	(Pittet et al., 2016; Casellas et al., 2020; Karadag et al., 2020; Zare et al., 2020; Vutcovici et al., 2021)
Social needs	Social health support systems and support groups (*n* = 15, 20.00%)	social support for work disability and daily activities, access to social workers and support groups, social health support (social isolation such as going to town, or not having access to toilets), Social support by health professionals, and self-help groups,	(Pittet et al., 2016; Schoultz et al., 2016; Britt, 2017; Cho et al., 2018; Kamp and Brittain, 2018; Casellas et al., 2020; Karadag et al., 2020; Khalil et al., 2020; Moon et al., 2020; Zare et al., 2020; Fawson et al., 2021; Long et al., 2021; Popov et al., 2021; Rubin et al., 2021; Volpato et al., 2021)
	Family/partner/ caregivers' support and involvement (*n* = 9, 12.00%)	sharing of medical information and disease symptoms with their life partner/ family, Social support from their family/partner, partner involvement,	(Viazis et al., 2013; Lahat et al., 2014; Cho et al., 2018; Kamp and Brittain, 2018; Khalil et al., 2020; Zare et al., 2020; Graffigna et al., 2021; Long et al., 2021; Volpato et al., 2021)
	Breaking down social isolation (*n* = 10, 13.33%)	breaking down isolation gaining support, Relationships and communication with others, To talk about the disease, contact with other people,	(Khan et al., 2016; Britt, 2017; Larsson et al., 2017; López-Sanromán et al., 2017; Wilburn et al., 2017; Sarwan et al., 2019; Zare et al., 2020; Cai et al., 2021; Fawson et al., 2021; Volpato et al., 2021)
Interpersonal intimacy needs	Intimacy support (*n* = 2, 2.67%)	Intimacy support	(Wilburn et al., 2017; Sarwan et al., 2019)
	Need related to pregnancy, and fertility (*n* = 1, 1.33%)	attention to Needs related to pregnancy and fertility,	(Volpato et al., 2021)
Practical needs	Health care access (*n* = 14, 18.67%)	Accuracy and Confidentiality of diagnosis, medication compliance, non-invasive disease monitoring, effective treatment support for IBD, More prevention and health promotion, Hygiene and Freedom from infection, Alternative Treatments, Access to care, Continuity of care (regular contact, and continuity facilitated the follow-up consultation),	(Bray et al., 2016; Lesnovska et al., 2017; Wilburn et al., 2017; Sarwan et al., 2019; Casellas et al., 2020; Karadag et al., 2020; Khalil et al., 2020; Al Khoury et al., 2021; Aluzaite et al., 2021; Goodsall et al., 2021; Graffigna et al., 2021; Long et al., 2021; Popov et al., 2021; Vutcovici et al., 2021)
	Facilities support (*n* = 4, 5.33%)	Availability of IBD care facilities (availability of their toilet, not having to share a room with others, quality of hygiene in hospitals, quicker appointments and diagnosis, and regular follow-up),	(Schoultz et al., 2016; Lesnovska et al., 2017; Jordan et al., 2018; Aluzaite et al., 2021)
	Financial support (*n* = 2, 2.67%)	legal support, Financial independence (Offer guidance and support navigating financial support through insurance, and government agencies), financial healthcare coverage,	(Cho et al., 2018; Popov et al., 2021)
	Occupational support (*n* = 9, 12.00%)	social support for work disability, understanding and flexibility with employment, Compatibility of the job with the disease, talking to the boss or coworkers about the disease, Work environment, Coordination between work and medical appointments, impact of UC on work, occupational health support (help with benefits and welfare when unable to work),	(Schoultz et al., 2016; López-Sanromán et al., 2017; Jordan et al., 2018; Casellas et al., 2020; Moon et al., 2020; Zare et al., 2020; Fawson et al., 2021; Popov et al., 2021; Vutcovici et al., 2021)
Daily living needs	Daily living tasks (*n* = 11, 14.67%)	social and practical support for daily activities, Talk about how illness is impacting daily activities (physical, comfort, safety, adequate nutrition, ability to carry out day-to-day activities, Transportation and parking at the hospital, need for assistance with household chores and childcare), concerns about diet, Exercise, and Leisure, need to help patients with routines,	(Khan et al., 2016; Schoultz et al., 2016; Britt, 2017; Wilburn et al., 2017; Cho et al., 2018; Sarwan et al., 2019; Casellas et al., 2020; Moon et al., 2020; Aluzaite et al., 2021; Long et al., 2021; Vutcovici et al., 2021)
	Disease management /self-care (*n* = 9, 12.00%)	provided self-management skills, self-care programs, develop individualized treatment plans,	(Berding et al., 2016; Wilburn et al., 2017; Sarwan et al., 2019; Casellas et al., 2020; Moon et al., 2020; Zare et al., 2020; Al Khoury et al., 2021; Cai et al., 2021; Fawson et al., 2021)
Patient-clinician communication needs	Dietician's support (*n* = 3, 4.00%)	assess and recommendations for nutrition during the routine visits, support from dieticians,	(Viazis et al., 2013; Schoultz et al., 2016; Volpato et al., 2021)
	Multidisciplinary care services/ holistic approach (*n* = 7, 9.33%)	Access to Multidisciplinary IBD Care Services, a holistic team approach,	(Schoultz, 2016; Lesnovska et al., 2017; Casellas et al., 2020; Moon et al., 2020; Graffigna et al., 2021; Popov et al., 2021; Vutcovici et al., 2021)
	Shared-decision making /patient-centered approaches (*n* = 10, 13.33%)	Patient-Centered Treatment Plan, involved in making treatment decisions,	(Politi et al., 2008; Cullen et al., 2010; Khan et al., 2016; López-Sanromán et al., 2017; Casellas et al., 2020; Al Khoury et al., 2021; Graffigna et al., 2021; Rubin et al., 2021; Volpato et al., 2021; Vutcovici et al., 2021)
	Technology supports (*n* = 6, 8.00%)	track symptoms and use of medication, improve the relationship by smartphone or online tools, Telehealth consultation, remote care,	(Niv et al., 2017; Casellas et al., 2020; Al Khoury et al., 2021; Goodsall et al., 2021; Rubin et al., 2021; Vutcovici et al., 2021)
	Information-sharing and good coordination between gastroenterologists, other specialists, and patients (*n* = 4, 5.33%)	coordination and information-sharing between gastroenterologists and other specialists, Access to Medical File Information, information-sharing between gastroenterologists,	(Pittet et al., 2016; Al Khoury et al., 2021; Fawson et al., 2021; Vutcovici et al., 2021)
	Support and patient-physician interaction and relationship (*n* = 11, 14.67%)	communicating with patients, interactions between patients and healthcare providers, positive relationships with clinicians, access to professional support, Honest and empathetic relationships with health professionals, Possibility for asking questions and doubts to clinicians, Respect and trust (understanding from HCP, sufficient time, and adequate help),	(Khan et al., 2016; Schoultz, 2016; Lesnovska et al., 2017; López-Sanromán et al., 2017; Casellas et al., 2020; Khalil et al., 2020; Cai et al., 2021; Fawson et al., 2021; Graffigna et al., 2021; Popov et al., 2021; Rubin et al., 2021)
	Easy access and contact with specialist staff (*n* = 4, 5.33%)	timely access to healthcare providers and clinic appointments, Access to GI Specialist/IBD Care/BD Nurse, Flexible appointment and follow-up schedule, Rapid and direct access, Accessible health professionals, The optimal time to talk to the doctor and/or nurse,	(Schoultz et al., 2016; Casellas et al., 2020; Karadag et al., 2020; Vutcovici et al., 2021)
	Immediate advice (*n* = 1, 1.33%)	quicker appointments and diagnosis and regular follow-up,	(Schoultz et al., 2016)
	Monitoring and follow-up (*n* = 4, 5.33%)	non-invasive disease monitoring, drug, symptom, and diet trackers, regular follow-up,	(Al Khoury et al., 2021; Cai et al., 2021; Fawson et al., 2021; Goodsall et al., 2021)
Support needs related to the future	Transitions needs (*n* = 1, 1.33%)	Transition needs	(Volpato et al., 2021)

#### 3.5.1. Psychological/emotional needs

IBD is, in fact, a disorder of the brain-gut axis. Considering that the health of the brain and gut are intertwined, patients with IBD predispose to developing anxiety and depression (Mikocka-Walus et al., 2016a). Resulting complications are reflected in this fact that IBD patients who suffered from anxiety and mental illness usually experience greater doses of steroids, escalation of therapeutics, disease flare ups, aggressive presentation, more hospitalizations, and higher risk of surgery (Bernstein et al., 2010; Ananthakrishnan et al., 2013; Mikocka-Walus et al., 2016b; Barnes et al., 2017; Kochar et al., 2018). Unfortunately, only a small fraction of IBD patients with anxiety and depression receive psychological or psychiatric needs (Bennebroek Evertsz' et al., 2012). Interestingly, using mental health support is not associated with gender, education, perceived disease course, or the severity of psychological distress. However, there is an inverse relationship between income and willing to engage in such supports (Knowles et al., 2018). Mental health issues are inevitably followed by higher health care costs, and hence, psychological screenings and relevant intervention generate significant savings (Lores et al., 2021). More works should be done in order to increase the acceptance of psychological interventions among IBD patients who need.

### 3.6. Consultation with patients with IBD and professional stakeholders

A total of 30 patients with IBD and 30 professional stakeholders (24 faculty members of the gastroenterology and hepatology group; 5 psychiatrists and faculty members of the psychology group and 1 medical informatics specialist) completed our survey. All experts confirmed the framework for the presentation of the findings of our review and made no recommendation for including any additional studies in the review.

Consistent with our review, experts emphasized the key role of patient education and support in managing IBD. They also noted that the factors might be enablers or barriers to the patient's quality of life depending on the situation. Additionally, they showed a lack of education and motivation and proper support as a barrier to improving the quality of life, and they insisted on the crucial role of addressing and acknowledging mental health issues before making lifestyle changes. Most patients with IBD emphasized stress, anxiety, high cost of medications, and lack of awareness about their situation and disease, which might reduce patients' quality of life and exacerbate their disease. Interestingly, our consultation was in line with the key point that paying attention to psychological support, obtaining physical and mental self-care, proper interaction of the patient with the therapeutic team, beneficial and accurate instruction, financial and insurance support, assessment of rare medications, and therapeutic team support are crucial.

## 4. Discussion

In the present scoping review, seventy-five studies assessing the information and supportive needs of patients with IBD were reviewed. Researchers in 62 and 53 studies reported information needs and supportive needs, respectively. Most of the information needs of patients with IBD reported in the studies were related to nutritional needs. Most patients also resorted to the health care team for the information they needed. According to the results of studies, patients with IBD considered educational needs to be the most important support needs.

Patients with IBD, including CD and UC, suffer from multiple concerns about the origin, long-term progression, and chronicity of their disease (Pittet et al., 2016). They also have ongoing stress owing to their condition. Although they strive to lead regular lives, the numerous complications of the disease prevent them from achieving this (Kemp et al., 2012). Furthermore, studies indicate that IBD sufferers experience financial, social, recreational, and health consequences leading to reduced health-related quality of life (Joachim, 2002; Larsson et al., 2017). Therefore, patients seek to gather information to improve the negative outcomes of IBD (Bernstein et al., 2011). The impact of having enough information on improving IBD management has been identified (Pittet et al., 2014). The most common sources of information for patients include gastroenterologists, the Internet, and general practitioners, respectively (Catalán-Serra et al., 2015).

Researchers in their studies many nations demonstrate that cultural and health system variations can have an impact on the needs of patients with IBD. According to the findings of a study on insurance coverage in various Asian nations, different countries have diverse insurance coverage rates. The problem may affect patients' needs for medical care. The rate of medical treatment was higher in nations with higher insurance coverage rates (Wei, 2016). It may be inferred from the findings of the aforementioned study and the different financial needs of the studies included in this scoping review that the healthcare systems and cultures of different nations can affect the needs of patients with IBD.

The nutritional needs of patients with IBD were found to be the most significant and often occurring needs in the studies included in this scoping review. Cultural variations in nutrition between nations may be one of the causes. The diet suggested for patients with IBD can differ depending on the country due to the problem. Hence, when educating patients with IBD, cultural dietary variances should be considered.

Researchers in one scoping review study (2021) investigated the care needs of patients with IBD in childhood, adolescence, and adulthood. The results of this study revealed that the main care needs in adults with IBD included information, medical, psychological, social, job-related, practical, future-related, and interactive needs (Volpato et al., 2021). Researchers in one systematic review revealed that patients with IBD lacked sufficient knowledge and information about medication (effectiveness, side effects, etc.,) (Santos et al., 2020). Contrary to the current study, researchers in prior review studies often examined only one aspect of the needs of patients with IBD; however, in this scoping review, the needs of patients with IBD have been thoroughly divided into three categories: informational, supporting, and psychological. As previously noted, the researchers in this scoping review showed that the most frequent supporting and informational needs were nutritional and educational needs, respectively. Based on the results of the studies and the current scoping review, it is crucial to consider the needs of patients with IBD due to the impact of this disease on their quality of life. Hence, health policymakers and managers can develop care and educational programs related to this disease in hospitals and health centers according to the needs of the patients. More focus should be placed on nutritional requirements, pharmacological side effects, surgical complications, and long-term implications of the disease in educational programs. Patients can simply obtain these educational programs by having them implemented online on Internet platforms.

As researchers in the present study showed, vaccination was one of the informational needs of patients with IBD. Researchers in another systematic review demonstrated that the rate of vaccination among patients with IBD was lower than desired, indicating a lack of knowledge and information about vaccination in the patients (Chan et al., 2021). In another review study, researchers examined the expectations of patients with IBD and showed that patients expected more information about the course of the disease, symptom control, and joint decisions with medical staff. Additionally, they also revealed that patients were referred to their gastroenterologists to obtain information about their disease (Al Khoury et al., 2021). Furthermore, they demonstrated that patients with IBD were referred to the health care team to compensate for the lack of knowledge and obtain information. According to the results of these studies, health professionals, particularly gastroenterologists, are the main sources of referral to acquire information on patients with IBD. Thus, gastroenterologists can take the lead in planning and educating the patients and share their decisions with them. Moreover, due to the significance of the patients' nutritional demands, nutritionists, as experts in this field, should share their perspectives with patients and fulfill their informational needs. Nurses play a key role in the management of patients with IBD, and their role includes assist in meeting their requirements by providing oral instruction and responding to their needs, monitoring of therapy, and continuous support. Health psychologists' roles is crucial in improving the quality of life and mental and physical health in patients with IBD. Finally, we maintain that multidisciplinary teams provide better management and care to patients with IBD.

The critical need for support in patients with IBD, according to studies in the current scoping review, is associated with educational, mental health, and social health needs. Hence, policymakers and health managers can meet the needs of the patients by developing educational and counseling sessions. It is also recommended that researchers in their future intervention studies should examine the impact of education on the quality of life, mental health, and social health in such patients.

## 5. Limitations

The present scoping review had certain limitations. Owing to the prevalence of co-morbidities with IBD, this study may not be generalized to all patients with IBD, since other co-morbidities were excluded from this study. In some studies, participants were selected through self-selection, and this may lead to heterogeneity of needs among participants. As with other scoping review studies, the qualitative review of the studies was not conducted due to their various methodologies. One of the limitations of the present study was the lack of access to the full text of some articles (12 articles), which may lead to unintentional bias. Finally, although a comprehensive search of the electronic databases was performed, not all related studies may have been entered.

### 5.1. Implications for future research

According to the results of the present scoping review, it is recommended that future studies should be founded based on different stages of the disease and patients' age. This classification facilitates the identification of the needs in each stage. It is also suggested that researchers in their future interventional studies should examine the impact of education on the quality of life, mental health, and social health in the patients.

### 5.2. Implications for clinical practice

Results can be used to illustrate inadequacies in the care of persons with IBD as well as to identify, prioritize, and design new healthcare systems. The needs discovered in this study can be utilized to develop effective treatments in order to address each need in clinical practice.

## 6. Conclusion

Overall, most of the information needs of patients with IBD reported in the studies were related to nutritional needs. In addition, most patients refer to the health provider team to obtain their information needs. According to the results of studies, patients with IBD regarded educational needs as the most supportive needs. The adoption of a comprehensive approach to care, which includes a positive relationship between the patient and the doctor, appropriate patient education, and prompt treatment of psychological diseases like depression and anxiety, is another suggested method to enhance the general quality of care and the psychological condition of patients. Hence, based on the requirements of these patients, health policymakers and managers can create care and educational programs concerning this disease in hospitals and health facilities. The primary referral sources for information on patients with IBD are medical experts, particularly gastroenterologists. Thus, gastroenterologists can take the initiative in organizing and teaching the patients and involve them in the decision-making process. Accordingly, it may improve communication and education to include well-designed written information or a website recommendation for doctor-patient sessions. For the active participation of patients as partners in their treatment, they must also be aware of the sources of patient information and make sure that they receive reliable information.

## Data availability statement

The original contributions presented in the study are included in the article/Supplementary material, further inquiries can be directed to the corresponding author.

## Author contributions

NN and HT: idea for the review, study selection, data extraction, interpretation of results, and writing of the manuscript. MF, SG, JS, AB, and SE: data extraction, interpretation of results, and writing of the manuscript. MK and MS: writing of the manuscript. All authors read and approved the final manuscript.
