# Supplementary material for: Identification of the informational and supportive needs of patients diagnosed with inflammatory bowel disease: a scoping review
Source: Front Psychol. 2023 May 11;14:1055449. doi: 10.3389/fpsyg.2023.1055449 (PMC10211349; doi:10.3389/fpsyg.2023.1055449)
Supplement: Supplementary file 1 [file Table_1.DOCX]

**Supplementary materials**

**Supplementary Table 1.** Inclusion and exclusion criteria.

| Inclusion criteria | | Exclusion criteria |
| --- | --- | --- |
| Population and sample | Only studies involving adult patients 18 years of age or older with a diagnosis of IBD including CD and UC (after the age of 18 years, excluding studies with a mixed population of children and adults). | - Other diseases. - Articles whose population was children and adolescents. |
| Study design | Primary research studies (no limitation to study design). | - Articles that have not been reviewed by peers - Articles that are only available in summary - Movies - News - Newspapers |
| Reporting | - Published studies - Dissertations - Conference papers and reports - Guidelines | - Books - Study protocol - Studies with no results or no full text available - Letter - News or magazine |
| Study focus | This study focuses on at least one of the following in people with IBD:   - Patients' information needs about IBD treatment and management. - Patients’ support needs about IBD treatment and management. - Patient’s psychological needs about IBD treatment and management. | - No reference to educational, supportive, informational, or psychological needs. - The information, educational, supportive, and psychological needs of people with IBD are limited to children or adolescents. - The information, educational, support, and psychological needs of mental health professionals, caregivers, or family members should be reported on behalf of participants. - Report medical, therapeutic, and pharmaceutical needs. - Evaluation of new tools, surveys, and questionnaires. |
| Study outcomes | Information, educational, supportive, and psychological needs of adults with IBD. | - Articles that merely describe the effectiveness of tools, treatments, medications, and training programs or interventions that the patient can use to manage IBD without a needs assessment, and that the patient's needs and wants are not addressed in his or her language or by physicians. - Studies that are exclusively on a specific population of IBD patients with specific medical conditions and do not include all patients with inflammatory bowel disease (IBD patients with cervical cancer, surgery, smokers only, veterans, needs related to transitional care, Ostomy, anal fistula surgery, colostomy, ileostomy, etc. or taking a special drug) were performed, removed. - We excluded studies that reported only unexplained consent, symptoms, or actions. |
| Publication date | Articles published from January 2000 to September 30, 2021. | Studies outside these dates. |
| Language | No language limitation. | - |
| setting | No setting limitation. | - |

**Supplementary Table 2.** Search strategy in PubMed/Medline

| Searches |
| --- |
| ("Inflammatory Bowel Disease*"[ti] OR "Inflammatory Bowel Diseases"[Mesh] OR “IBD”[ti] OR “Crohn Disease”[ti] OR “Crohn's Disease”[ti] OR “Crohns Disease”[ti] OR "Crohn Disease"[Mesh] OR “Ulcerative Colitis”[ti] OR “Colitis, Ulcerative"[Mesh])  AND  ( “Needs Assessment*”[tiab] OR Need*[tiab] OR "Needs Assessment"[Mesh] OR “patient need*”[tiab] OR “Patient Preference*”[tiab] OR “Patient Attitude*”[tiab] OR “Patient Expectation*”[tiab] OR "Patient Preference"[Mesh] OR “Educational Needs Assessment”[tiab] OR “Educational Need*”[tiab] OR “Patient Education”[tiab] OR “Education of Patients”[tiab] OR "Patient Education as Topic"[Mesh] OR “Information* need*”[tiab] OR “Consumer Health Information”[tiab] OR “Information Seeking Behavior*”[tiab] OR “information seeking”[tiab] OR “information source”[tiab] OR “Supportive need*”[tiab] OR “Support* need*”[tiab] OR “psychological need*”[tiab] OR “Mental health needs”[tiab])  AND  2000/01/01:2021/09/30[dp] |

**Supplementary Table 3.** Basic characteristics of the included studies in this systematic review.

| **First Author/year** | **Country, study participants, sample size, recruitment setting** | **Aims of the study** | **Study design** | **Method of data collection** | - **Outcome (measures)** | **IBD patient type n (%),**   - **Disease duration** | - **Kind of Identified needs** | **Key findings as reported in the paper** |
| --- | --- | --- | --- | --- | --- | --- | --- | --- |
| **O'Sullivan *et al.,* 2000**  **(95)** | Ireland  N=212(IBS+IBD)  UC  Age mean =41years  51% male (42)  49% female (40)  CD  Age mean = 38years  32% male (19)  68% female (41),  gastroenterology outpatient clinic | - Identify the information needs, levels, and associated factors in IBS, and contrast this with patients with IBD. | Comparative study | Questionnaire | - Knowledge (A simple visual analog scale/VAS) Satisfaction (A simple visual analog scale/VAS) - Patient educational needs (open-ended Questionnaire) - Anxiety And Depression (HAD scale) | - UC 82 (57.74%) - CD 60 (42.26 %), - "Ulcerative colitis: - the median duration of the disease was 5.3 years (range 0.1 to 42.2 years), Crohn’s disease: - the median duration of disease was 7.4 years (range 0.4 to 29.1 years)" | - Information needs - Support needs | The result of the study showed that most IBS patients feel insufficiently informed, particularly about the risk of serious disease and the role of diet. |
| **Quan *et al.,* 2003**  **(94)** | USA  -  N=743,  local newspaper,  Television and radio interviews with the local panelists,  mailed brochures of the workshop to gastroenterologists and physicians for distribution at their clinics | - The knowledge of IBD of participants attending educational workshops offered to the public. - The effect of the workshop on participants’ knowledge level. | Quasi-experimental study | survey | Disease-related knowledge (CCKNOW) (self-administrated Crohn’s and Colitis Knowledge Score questionnaire) | - UC (N/A)   CD (N/A) | - Information needs - Support needs | The public’s general knowledge of IBD is low. Educational programs oriented toward IBD improve participants’ knowledge, and the knowledge they acquired is retained for at least 3 months. |
| **Ryan *et al.,* 2003**  **(93)** | UK  N=312  Age mean = 49.2 (20 to 83)  37% male (116)  63% female (196),  Department of Surgery University Hospital | Assessed awareness among patients with a CD of the risks   - of smoking on their disease. | Cross-sectional study | Online self-administered  questionnaire | Awareness of the risk of smoking on CD (Self-administered questionnaire) | - CD (100%) | - Information needs - Support needs | This study demonstrates the need for more patient education concerning the particularly harmful effects of smoking on CD. |
| **Rezailashkajani *et al.,* 2006**  **(92)** | Iran  N=100  Age mean =36 (18 over 50 years)  36 % male (36)  64% female (64),  a referral outpatient gastro  enter ology  clinic in central Tehran | - Assessment level of self-rated knowledge of the disease among IBD patients.   Determining the health information preferences of Iranian IBD patients. | Cross-sectional study | Questionnaire | - Disease-related knowledge (CCKNOW) (self-administrated Crohn’s and Colitis Knowledge Score questionnaire) | - UC 86 (86%) - CD 9 (9%) - IBDU 5 (5%) - the median duration of the disease was 4 years | - Information needs | Despite the overt inclination of Iranian IBD patients to know more about their disease, their knowledge levels were significantly lower than the IBD patients in developed countries.  The more profound knowledge deficit in IBD complications may lead to disastrous aftermaths such as late diagnosis of colorectal cancer induced by prolonged IBD.  Vigorous patient education programs for Iranian IBD patients are suggested to focus on areas of knowledge.  Favorite disease-related knowledge topics by patients’ sex and type of IBD. |
| **Politi *et al.,* 2008**  **(91)** | Switzerland  N=728  Age mean= 30% were less than 35 years old  47.5% male (346)  52.5% female (382),  cohort | Evaluate the methods being used for communication of information about their disease to a multi-national IBD cohort, as well as patient's preferences concerning current and future sources of information, including electronic media and patients' associations, and their level of satisfaction with the information provided. | Cohort study | Internet-based questionnaire | - Information-seeking behavior (Questionnaire) - Degree of satisfaction with the data provided (Questionnaire) - Patient's preference for particular forms of media (Questionnaire) | - UC 617 (% 66.6) - CD 296 (%33.4) - IBDU 4 (%0,4) | - Information needs - Support needs | There should be greater roles for IBD patients' associations and international IBD-research organizations, and increasing use of electronic media. |
| **Cullen *et al.,* 2010**  **(90)** | Ireland  N=100  Age median = 37 (29–49) years  51% male (51)  49 % female (49),  gastroenterology clinics | This study aimed to assess knowledge of medication safety and attitudes toward a broad variety of treatments commonly used in patients with IBD. | Cross-sectional study | Survey  Questionnaire | - Visual analog scale (VAS) relating to the safety of commonly used IBD treatments and assess general attitudes to particular medications and included elements relating to effectiveness as well as safety. - The questionnaire to rank information sources. - Harvey–Bradshaw index (HBI) for CD disease activity and Mayo score for UC disease activity | - UC 44 (%44) - CD 56 (56%) - mean disease duration was 7 (3–9) years. | - Information needs - Support needs | The result of the study showed that IBD patients want to be informed of all potential adverse events and identify their gastroenterologist as their principal information source. |
| **Molnár *et al.,* 2010**  **(89)** | Hungarian  Age median = 37 (29–49) years | Evaluate the most common topics discussed by the patients and the Internet doctor on one of the most popular Hungarian IBD websites. | Qualitative study | Online questions about IBD. | Online questions about IBD | - UC (36.1%) - CD (39.4%) - IBDU (24.5%) | Information needs | The result of the study showed that women ask questions on the Internet significantly more frequently than male patients. Also, the vast majority of the questions asked by the IBD patients, regardless of gender and disease type, were related to the medication. |
| **Subasinghe *et al.,* 2010**  **(88)** | Sri Lanka  N=184  Age mean =44.5 years (range 20–78)  45.1% male (83)  54.9 % female (101),  outpatient gastroenterology clinics of a tertiary care hospital | This study aimed to analyze the deficits in knowledge for future health education programs. | Cross-sectional study | Survey  Questionnaire | - Disease-related knowledge (CCKNOW) (self-administrated Crohn’s and Colitis Knowledge Score questionnaire) | - UC (83.2%) - CD (16.8%) - The mean duration of IBD was 8.17 | Information needs | There is a lack of knowledge regarding colorectal cancer risk and surgical interventions.  There was no significant difference in the knowledge scores between genders but there was a significant association with the educational level. |
| **Bernstein *et al.,* 2011**  **(87)** | Canada  N=74  Age mean = 37.8  47% male (35)  53% female (39)  five gastroenterology practices including one specialized IBD clinic, one hospital-based  general gastroenterology clinic, and three communities-based gastroenterology practices. | Assess the information needs and experiences of patients who were recently diagnosed   - with IBD. | Cross-sectional study | Questionnaire | - Disease status: Manitoba IBD Index (MIBDI) - Inflammatory Bowel Disease Quality of Life: IBD Quality of Life Index (IBDQ) - Information needs: A survey with two series of questions - Patients seeking information in a healthcare setting: Krantz Health Opinion Survey (HOS) | - UC 38 (51.3%) - CD 34 (46 %) - IBDU 2 (2.7 %) | - Information needs - Support needs | The result of the study showed that most of the participants did not receive the information they considered important during the first 2 months following their diagnosis.  Participants preferred to be educated about their disease by physicians. |
| **Conrad *et al.,* 2012**  **(86)** | Germany  N=1056  Age mean = 42 years  35% male (368)  65 % female (688),  specialized gastroenterological practices, university outpatient clinics, and the member registry of the relevant patient organization DCCV and via advertisements | - Evidence-based and consented pathways for patients with IBD. - Call for tailored education programs to foster shared decision-making and patient self-management. | Cross-sectional study | Questionnaire  Survey | - Anxiety and depression: (HADS) - Information needs: (Questionnaire) - Preferred information sources: (Questionnaire) | - UC (42%) - CD (58%) - 0 – 3 years (%13.8) - 4 – 6 years (%15.9) - 7 – 9 years (%15.6) - 10 and more years (%54.8) | Information needs | The result of the study showed that the high information needs of IBD patients may serve in the planning of future educational programmers. |
| **Wong *et al.,* 2012**  **(13)** | Canada  N=271  Age mean = 46.50 (SD= 14.50)  39% male (106)  61% female (165),  population-based research registry(cohort) | Identify the information needs and preferences of persons with longstanding IBD. | Cohort study | Questionnaire | - Disease status (MIBDI) - Disease-related quality of life (IBDQ) - Patients' experience when they found out they have IBD (Questionnaire) - Information needs (Questionnaire) | - UC 138 (51%) - CD 132 (48.7 %) - IBDU 1 (0.36 %), - mean disease duration 11 years | - Information needs - Support needs | Approximately 10 years after diagnosis, only a small percentage of persons with IBD believed they received the correct amount of information about the issues they regarded as most important to have discussed at diagnosis. |
| **Yeung *et al.,* 2012**  **(85)** | Canada  Physicians:  N= 43  Men= 27.90%  Women= 72.10%  Patients:  N= 167  Men= 38.92%  Women= 61.08%  Age= 46.50,  Zeidler Gastroenterology Health Centre at the University of Alberta Hospital | Investigating and describing knowledge and values regarding immunization among IBD patients and gastroenterologists. | Cross-sectional study | Questionnaire | Information needs (Questionnaire) | - UC (40.12%) - CD (59.28%) - IBDU (0.60%) - The mean duration of the disease was 14 years | - Information needs - Support needs | Gastroenterologists have limited knowledge of their IBD patients’ immunization status and rarely take an adequate immunization history.  Substantial proportions of IBD patients and gastroenterologists lack adequate knowledge of established immunization guidelines before the initiation of immunosuppressive therapy. |
| **Huang *et al.,* 2013**  **(84)** | Canada  N=299  Age mean =36.5 years  44% male (60)  56% female (76),  gastroenterology clinics | Assess the IBD patients' awareness of complications related to their bowel disease,  and where they obtain their information, to identify gaps in patient education that may benefit from educational programs. | Cross-sectional study | Survey | patients' awareness AND educational gaps: (questionnaire) | - UC 104 (%35) - CD 177 (%59) - mean disease duration was 11.40 | Information needs | The result of the study showed that most IBD patients are aware of extraintestinal manifestations that are either more prevalent or heavily emphasized (e.g., cancer).  They are deficits in knowledge, particularly of life-threatening systemic complications such as venous thromboembolism. |
| **Mukewar *et al.,* 2013**  **(83)** | USA  N= 93,  YouTube, is one of the most popular websites in the United States. | Assess the source, content, and accuracy of the most commonly viewed YouTube videos related to IBD and gather and assess the demographic data of the viewers | Cross-sectional study | Checklist | - how IBD patients obtain healthcare-related information and whether the information is accurate (Questionnaire) | - N/A | Information needs | The YouTube videos on IBD assessed in this study were predominantly viewed by middle-aged adults.  Most of the videos were uploaded by patients or pharmaceutical companies and had a negative bias toward conventional treatments.  IBD-related education content on YouTube is mostly poor in quality.  Videos discussing alternative treatments were uploaded by patients or pharmaceutical companies and were greater in number than patient education videos.  Healthcare professionals and professional societies need to counteract the incorrect “education materials” on the Internet, particularly for the targeted population. |
| **Selinger *et al.,* 2013**  **(82)** | Australia  N=145  Age median= 32years (18–45)  100 % female,  tertiary IBD outpatient clinics and offices | The study aimed to assess patients' views on IBD and pregnancy and to Evaluate any association with subject knowledge. | Cohort study | Survey | - Knowledge of pregnancy-related issues in IBD: (CCPKNOW questionnaire) - Attitude (researcher-made attitude assessment tool) | - UC 65 (%44.8) - CD 65 (%44.8) - IBDU 15 (%10.4) - "0–5 years: 42 (%29.0) - >5 years: 103 (71.0)" | - Information needs - Support needs | The association between poor subject knowledge and views may be detrimental to the successful delivery of a healthy child by patients with IBD.  Improved education programs relating to the impact of IBD on pregnancy are vital, not just when considering conception, but for all young women early after disease diagnosis, to allow patients to make informed decisions about having children.  Identification of patients with poor knowledge, using tools such as CCPKNOW, will allow the targeted education and counseling of women at particular risk, which may in turn improve clinical outcomes for both mothers and babies. |
| **Viazis *et al.,* 2013**  **(81)** | Greece  N=1181  Age mean= 31% of patients are between30-39 years  50% male (594)  50% female (587),  at the outpatient clinic | This study aimed to identify inflammatory bowel disease patients’ perspectives regarding everyday life issues. | Cross-sectional study | - Questionnaire | - Patients’ social life and their disease-related emotional status (questionnaire). - Patients’ knowledge and information-seeking behavior regarding IBD (questionnaire). - Conditions and relationships in the work environment (questionnaire). - Explored patients’ perceptions about the support they receive from their family and friends (questionnaire). | - UC 539 (46%) - CD 642 (54%) - <1 (12%) - 1-5 (36%) - 6-10 (26%) - 11-15 (11%) - 16-20 (7%) - >20 (7%) | - Information needs - Support needs | Greek IBD patients seek a better organized medical and hospital environment, a tighter relationship with their physician leading to a deeper understanding of their emotional and social status, and also the opportunity to receive more disease-related information and a broad educational program for those responsible for human resources in their working environment.  The vast majority of our patients reported having overwhelming support from their relatives, without which they would not be able to cope with the burden of their disease.  Patients needed more thorough information regarding complications of the disease (especially cancer) and treatment-related adverse events. |
| **Greveson, 2014**  **(80)** | UK  N=136  Age median = 38 years (range 18–85 years)  44% male (60)  56 % female (76),  hospital outpatient clinic | Explore the IBD patient’s experience of travel, including pre-travel preparation, and examine the availability of travel information, to evaluate and improve the quality and provision of pre-travel advice. | Cross-sectional study | Survey and Questionnaire | - demographics and disease catachrestic: (self-administered survey)   experience about travel with IBD: (self-administered survey) | - UC 69 (%51) - CD 67 (%49) | Information needs | The result of the study showed that IBD Passport was developed as a non-profit, IBD-specific travel resource, aimed at providing evidence-based information for IBD patients and health professionals. |
| **Lahat *et al.,* 2014**  **(79)** | Israel  N=101  Age mean= 45.36 years  52.4% male (53)  47.6 % female (48),  Medical Center | This study aimed to assess patients’ views regarding sharing of information with their partners. | Cross-sectional study | Questionnaire | sharing IBD patients’ health problems with their partner, partner involvement in disease management, and their views on partner involvement and how it should be achieved: (questionnaire) | - UC 39 (%38.7) - CD 62 (%61.3) - mean disease duration was 9.6 | - Information needs - Support needs | The result of the study showed that most IBD patients in this study wanted their partners to be more involved with their health problems and believed that greater partner involvement could help them deal better with their disease. |
| **Lesnovska *et al.,* 2014**  **(78)** | Sweden  N=26  Age median= 62 years (19-76 years)  54% male (14)  46% female (12),  outpatient clinic | This study aimed to explore the need for knowledge as expressed by patients diagnosed with IBD. | Qualitative study | Interview | - Explore the knowledge needs of patients suffering from IBD: (interview) | - UC 12 (%40) - CD 18 (%60) - 0–5 years since diagnosis 8 (%27) - >5–10 years since diagnosis 4 (%13) - >10–15 years since diagnosis 6 (%20) - >15 years or more since diagnosis 12 (%40) | - Information needs - Support needs | The result of the study showed that knowledge needs were related to what to expect when living with IBD to manage everyday life.  There was a great variation in the need for knowledge, which was greatest immediately following diagnosis and during relapse. |
| **Shepherd *et al.,* 2014**  **(76)** | England  N=260 (patients and healthcare professionals)  Patient=136  Age median-patient = 38 years (range 18–85);  44% male (60)  56 % female (76)  HCP=105  50% male  50% female,  outpatient clinic, | This study aims to explore the experience of travel, and pretravel preparation was undertaken by the patient with IBD and examine IBD healthcare professionals (HCP) confidence in providing travel advice and the content of that advice. | Cross-sectional study | - Survey | - demographics and disease catachrestic: (self-administered survey) - Experience about travel with IBD: (self-administered survey) | - UC 69 (%51) - CD 67 (%49) | Information needs | Having a diagnosis of IBD limits travel for many patients.  IBD patients should be encouraged to seek adequate pre-travel advice and signposting to relevant resources.  Effective communication and joint travel consultations between the IBD team and travel health specialists are advised.  Individuals receiving immunosuppressant medications should be adequately counseled regarding vaccinations, particularly the need to avoid ‘live’ vaccines. |
| **Becker *et al.,* 2015**  **(75)** | Canada  N=313  Age mean =55% were between 20–29 years  23% male  77% female,  CCC website and pamphlets in the gastroenterology outpatient clinics at the University of Alberta Hospital and the University of Calgary. | To assess the impact of IBD on patients and families concerning leisure, relationships, mental well-being, and financial security, and to evaluate the quality and availability of IBD information. | Mixed-method study | - Focus group   online survey | - The impact of IBD on their lives: (Online survey) - health literacy/information needs: (Online survey) | - UC (32%) - CD (62%) - IBDU (6%) | Information needs | The impact of IBD on interpersonal relationships and leisure activities was significant among IBD patients and their families.  Understanding the disease and alternative treatment options, was of high interest.  Respondents had a strong desire to obtain information regarding disease progression, especially extraintestinal symptoms. |
| **Burkhalter *et al.,* 2015**  **(74)** | Switzerland  N=230  Age median = 40 (range 18-85)  59.6% male (137) 40.4% female (93),  Swiss IDB cohort | Assess IBD patients’ needs. | Mixed-method study | Cohort study  Structured interviews | - The SF-36 measures patient health and quality of life. - The HADS determines the levels of anxiety and depression. - The IBDQ for quality of life in IBD patients.   Patient need: Structured interview | - UC 112 (%48.7) - CD 118 (%51.3) | Support needs | Their results have provided an understanding of the patient’s needs, problems, and the current nursing role in caring for IBD patients.  According to experts’ interviews, the consensus statements are found mostly relevant with most items not yet carried out. |
| **Catalán-Serra *et al.,* 2015**  **(12)** | Spain  N=379  Age mean = 37.9 years (16-76)  50.1% male (190) 49.9% female (189),  outpatient gastroenterology clinics | The objective of the study was to determine the information resources these patients used, together with their perceived information gaps and expected preferences. | Cross-sectional study | semi-structured question survey | - Semi-structured questionnaires in three aspects: demographic and disease-related data, questions on how patients obtained information about their disease, and questions relating to future information preferences. | - UC (43%) - CD (57%) - Patients with a disease duration of fewer than two years (23.1%) | Information needs | The result of the study showed the importance of gastroenterologists as information sources, the growing importance of the Internet as a reference tool for information about the disease, and the future needs and preferences of our IBD patients. |
| **Yoo *et al.,* 2015**  **(73)** | Korea  N=313  69.0% were below the age of 40 years  59.4% male (186) 40.6% female (127),  outpatient gastroenterology  clinic of a tertiary care hospital in Seoul  (from September 2011 to January  2012) | Identify related knowledge and information demands of patients with IBD. | Cross-sectional study | Questionnaire | - Crohn’s and Colitis Knowledge (CCKNOW) - Information Needs (crafted - through literature review) | - UC 144 (46%) - CD 169 (54 %) - 6 years(62.3% of participants) | Information needs | The effectiveness of the training and education given to patients can be maximized in this education system when the information about disease and medications for CD patients or information about disease and diet for colitis patients is primarily provided according to the degree of the patient’s need for information. |
| **Berding *et al.,* 2016**  **(72)** | Germany  N=181  Age mean =39.9 years  31% male (56)  69% female (125),  German Crohn’s and Colitis Association by sending study information to self-help groups, DCCV members, interested patients all over Germany, and gastroenterologists were asked to inform their IBD patients about the study. | A manualized education program for IBD patients. | RCT | Survey  Questionnaire | - A modified validated German version (IBDPC) of the Rating Form of the - IBD Patient Concerns questionnaire - Fear of progression was assessed using the Fear of Progression Questionnaire-Short Form (FoP-Q-SF) - The Health Education Impact Questionnaire (heiQ) - Perceived disease activity was measured using the German Inflammatory Bowel Disease Activity Index (GIBDI) - HRQoL (SF-12 questionnaire) - The Patient Health Questionnaire-4 (PHQ-4) was used as a screening instrument for depression and anxiety. - To assess the use of coping strategies, participants were asked which of the coping strategies from a list of 23 items they applied. - The participants’ satisfaction with the program was evaluated using 5 items. | - UC (45%) - CD (55%) - the mean duration of the disease was 10.2 years | - Information needs - Support needs | The result of the study showed that education programs contributed to improvements in psychological distress, self-management skills, and coping and were appreciated by its attendees. |
| **Bray *et al.,* 2016**  **(71)** | Canada,  -  The objective was to gather a small group  of patients and healthcare professionals to share personal stories of the “real life” challenges of living with IBD | Obtain a deeper understanding of the unmet needs of IBD patients and their caregivers to increase public awareness, develop advocacy strategies, and identify research priorities. | Qualitative study | Interview | - N/A | - N/A | Support needs | Through personal vignettes, patients articulated a pressing need to increase understanding of the challenges faced by people suffering from IBD among both healthcare professionals and the general public,  develop best practices for navigating life transitions and addressing the unique challenges faced by children with IBD, and provide equitable access to appropriate, effective, and affordable treatments. |
| **Greveson *et al.,* 2016**  **(70)** | N=260 (patients and healthcare professionals)  Patient=132  Age median-patient = 38 years (range 18–85);  45% male (60)  55 % female (72)  NHCP=128  50% male  50% female,  IBD attending an outpatient clinic, HCPs recruited using regional and international network databases. | - This study aims to explore the experience of travel, and pretravel preparation was undertaken by the patient with IBD and examine IBD healthcare professionals (HCP) confidence in providing travel advice and the content of that advice. | Cross-sectional study | Survey | - demographics and disease catachrestic: (self-administered survey) - experience about travel with IBD: (self-administered survey) | - UC 65 (%49) - CD 67(51%) | Information needs | The result of the study showed that the majority of patients with IBD feel their disease affects both travel in general and destination; however, surprisingly despite some concerns, patients still travel abroad even if they have suffered a recent flare and few seek prior expert medical advice. |
| **Khan *et al.,* 2016**  **(69)** | Caucasian, Black/African American, Hispanic  N=15  Age = 33.3% of patients greater than 46 years  33.3% male (5)  66.7 % female (10),  Mount Sinai Crohn’s and Colitis Registry | - This study was designed to explore the thoughts of IBD patients regarding: - The major hurdles of living with IBD. - The information needs of patients with CD or UC.   How application technology can be used to improve patient quality of life. | Qualitative study | - Focus group | - Patient's quality of life: (The short IBD Questionnaire (SIBDQ)) - Health information needs and technology platforms that could meet these needs: (Interview with a focus group) | - UC - CD | - Information needs - Support needs | The result of the study showed that IBD patients need mobile health technologies that evaluate disease control and the goals of care.  Patients feel an objective assessment of their disease control, goal setting, and physician feedback will greatly enhance the utilization of all mobile health applications. |
| **Pittet *et al.,* 2016**  **(68)** | Switzerland  N=728  Age mean = 30% were less than 35 years old  47.5% male (346)  52.5% female (382),  previous study participants  (Swiss Inflammatory Bowel Diseases Cohort (SIBDC) Study) | Assessing information-seeking activity, sources, and topics of information among patients suffering from IBD in Switzerland. | mixed-methods study | - Semi-narrative survey - Focus groups   Cohort data | - Information needs and expectations of patients - Anxiety and depression (HADS) - Perceived stress (PSQ) - Global social support (ESSI) - The general quality of life (SF-36) - Physical Component Score (PCS) - Mental Component Score (MCS) - Disease-related quality of life (IBDQ-32)   Post-Traumatic Stress Diagnostic symptoms (PDS) | - UC 321 (44.1%) - CD 407 (55.9%), - one quarter had a disease duration > 15 years | - Information needs - Support needs | The study results showed that information remains insufficient for IBD patients on many aspects of their disease. |
| **Schoultz, 2016**  **(67)** | Scotland,  snowball sampling methods | To develop and collate the evidence for a definitive randomized controlled trial (RCT) testing the effectiveness of MBCT  for patients with inflammatory bowel disease (IBD). | Mixed-method study | review articles survey | Perspective and experience of current IBD services and recommendations for future (survey)  Quality of life (survey) | N/A | Support needs | The findings from the first three publications highlighted the disease-related concerns and psychological needs of patients with IBD. The findings from the last three publications highlighted how feasible it is to use MBCT in IBD and emphasized the IBD patients’ perspectives on MBCT.  Faced with the evidence about the need for psychological support for patients with IBD and other anecdotal evidence, it was important to examine if the experiences described in the literature were similar for IBD patients in Scotland. |
| **Schoultz *et al.,* 2016**  **(66)** | Australia  N=767  Age= N/A  47.07% male (361) 52.93% female (406),  purposive and snowball sampling methods | To explore IBD patients’ experiences of current services and make recommendations for future service development | Qualitative study | Survey | Patients’ experiences: (survey) | N/A  "Length of diagnosis  Less than a year 17 (2.33)  1–5 years 265 (36.35)  5–10 years 181 (24.82)  10–20 years 142 (19.47)  Over 20 years 124 (17.00)" | Support needs | Three key themes emerged:  Quality of life: Participants highlighted the impact the disease has on quality of life and the desperate need for IBD services to address this more holistically.  IBD clinicians and access: Participants recognized the need for more IBD nurses and gastroenterologists along with better access to them. Those with a named IBD nurse reported being more satisfied with their care.  An explicit IBD care pathway: Patients with IBD identified the need of making the IBD care pathway more explicit to service users. |
| **Sephton *et al.,* 2016**  **(65)** | UK  N= 8  Age mean=42.37 (SD= 12.07)  50% male (4)  50 % female (4),  at the education program | This qualitative study aimed to evaluate the impact of providing a group patient education program on the psychosocial elements of living with IBD. | Qualitative study | semi-structured qualitative interviews | Educational needs (semi-structured interview) | - UC (62.50%) - CD (37.50%) - mean disease duration was 6.37 | - Information needs - Support needs | This study demonstrated the positive impact of providing group education on the psychosocial aspects of living with IBD.  New themes previously unreported identified and demonstrated that providing group education addresses the gap in knowledge provision, which is unable to be achieved through traditional outpatient consultation.  The implementation of a group education program offers patients not just knowledge but interaction with others and a supportive learning environment, which has the potential to positively impact IBD care. |
| **Wheat *et al.,* 2016**  **(64)** | USA  N=28 (patients and providers)  Patient=18  Age mean-patient = 31.6 years  61.1% male (11)  38.9 % female (7)  Provider=10  50% male (5)  50% female (5),  medical university, Providers were identified through publicly available online directories and contacted via email and Patients were identified through their providers | - The primary aim of this study is to identify educational needs and barriers and factors associated with non-adherence among inflammatory bowel disease (IBD) patients. | Qualitative study | Semi-structured interviews | - Adherence (interview) - Information needs (interview) | - UC - CD - mean disease duration was 8.60 | Information needs | Their findings highlight several deficits in knowledge in IBD patients.  They identified factors associated with IBD patient comprehension, decision-making, and non-adherence to therapy.  These results can be used to develop targeted educational resources to improve adherence among IBD patients. |
| **Britt, 2017**  **(63)** | USA | - Use an existing taxonomy that had previously been developed and used in other social support contexts.   Examine the functions of an online social support group for CD and UC, so using an existing taxonomy in a modified form could potentially account for the variety of support messages throughout the group. | Qualitative study | N/A | N/A | - UC - CD | Information needs | The findings from the content analysis revealed that informational support was the most sought and shared by group members, followed by emotional support.  Symptom management and remission of CD and UC were among the most frequently occurring messages within the forum.  Group members shared successful treatments that kept the symptoms of CD and UC managed, and offered information to fellow group members.  For those in remission, group members shared treatments that they thought contributed to remission and offered recommendations to fellow group members. |
| **Larsson *et al.,* 2017**  **(62)** | Sweden  N=15  Age mean =50 years (range 29–63)  40% male (6)  60 % female (9),  Gastroenterology department at a university hospital | To examine disease-related stress, coping strategies, and the need for information and support in patients with IBD. | Qualitative study | Interview | Interview with IBD patients to examine: disease-related stress, coping strategies, and the need for support in patients with IBD. | - UC 7 (%47) - CD 8(%53) - 5 patients had a disease duration of ≤ 5 years, - and 10 of > 5 years. | - Information needs - Support needs | Fecal urgency and the fear of losing bowel control are important stressors for patients with inflammatory bowel disease. The patients handle this problem using various coping strategies depending on the type of stressful events. Both instrumental and emotional support were requested which primarily occurred at the time of diagnosis and disease flare-ups. |
| **Lesnovska *et al.,* 2017**  **(61)** | Sweden  N=26  Age median= 62 years (19-76 years)  54% male (14)  46 % female (12),  outpatient clinic | This study aimed to explore the perceptions of healthcare among persons living with IBD. | Qualitative study | Focus group | perception of healthcare among IBD patients: (Semi-structured interview) | - UC (61.54%) - CD (38.46%) | - Information needs - Support needs | The findings show the importance of establishing a respectful and trusting relationship, facilitating healthcare staff and persons with IBD to work as a team in fulfilling individual care needs. |
| **López-Sanromán *et al.,* 2017**  **(60)** | Spain  N=436  Age median= 46.2 years  52.8% male (229)  47.2 % female (205),  hospital gastroenterology clinics | The UC-LIFE survey aimed to evaluate the perceived everyday and emotional impact of UC on patients attending outpatient clinics in Spain and explored patient-physician communication. | Cross-sectional study | Survey  Questionnaire | - perceived every day and emotional impact of UC patients: (UC-LIFE was a cross-sectional survey) | - UC (100%) - The median duration of UC was 8 years | - Information needs - Support needs | Findings support the need for a more patient-centered approach to the care of UC patients, including psychological, emotional, and social aspects. |
| **Niv *et al.,* 2017**  **(59)** | Israel  N=105  Age mean= 34.2 (SD=10.7) years  49.5% male (52)  50.5 % female (53),  IBD clinic at the Rabin Medical Center | The objective of the present study was to examine the association between level of certainty, self-epistemic authority, Internet information-gathering habits, and health-related quality of life. | Cross-sectional study | Questionnaire | - health-related quality of life: (SIBDQ questionnaire) - level of certainty: (Mishel Uncertainty in Illness Scale (MUIS)) - self-epistemic authority: (questionnaire developed and validated by Raviv et al.)   seeking information about Crohn’s disease via the Internet.: (questionnaire) | - CD (100%) | - Information needs - Support needs | The result of the study showed that the level of certainty proved an important variable associated with health-related quality of life in CD patients. |
| **Restall *et al.,* 2017**  **(58)** | Canada  N=45  Age mean =45.4 years (21 - 73)  49% male (22)  51% female (23),  Manitoba IBD Cohort Study | - How do people with IBD engage with disease-related information for daily health decision-making? | Qualitative study | Interview | Disease status: (MIBDI) | - UC 26 (58%) - CD 19 (42%) - the mean duration of the disease was 10.9 years | - Information needs - Support needs | Findings illustrate the changing need for health-related information for IBD, and with evolving health and life circumstances.  Practitioners can be responsive to the information needs of people with IBD by having high-quality information available at the right time in a variety of formats and by supporting the incorporation of information in daily life |
| **Wilburn *et al.,* 2017**  **(57)** | UK  N=30  Age mean =47.9  40% male (12)  60% female (18),  Crohn’s registry at the Manchester Royal Infirmary Hospital. | - The study aimed to understand how the lives of individuals with CD are affected. - A secondary aim was to generate a pool of potential items for the Crohn’s Life Impact Questionnaire (CLIQ). | Qualitative study | Unstructured in-depth qualitative interviews | Psychological needs: (interview) | - CD - the mean duration of the disease was 14.3 years | Support needs | The CD has a major impact on the need-fulfillment and, consequently, the QoL of patients. |
| **Cho *et al.,* 2018**  **(56)** | Canada  N=21 (Pediatric and Adult)  Age mean = 37.6 (18-30)  38 % male (8)  62% female (13),  McMaster University  Medical Centre adult infl amatory bowel disease (IBD) clinic | - Identify the needs of young adults with IBD. | Qualitative Study | Semi-structured interviews  (By telephone or in person) | - The patient needs (Semi-structured interview) | - UC 2 (10%) - CD 19 (91%) | - Information needs - Support needs | The result of the study showed that the most commonly reported needs were psychosocial and the least common was daily living needs. |
| **Jordan *et al.,* 2018**  **(55)** | UK  N= 25  Age mean=36.50  44% male (11)  56 % female (14),  IBD clinical service | - Investigate the specific situations, thoughts, perceptions, appraisals, beliefs, and behavior that people with IBD describe as linked to their symptoms of anxiety and low mood. - Explore the type of psychological help or support people with IBD and elevated symptoms of anxiety and low mood would like as part of routine medical care. A secondary aim was to consider our findings of the knowledge associated with the competencies set for psychological therapists delivering interventions for long-term conditions. | Mixed-method study | - Semi-structured face-to-face interviews - Questionnaire | - Anxiety (generalized anxiety disorder 7 measure (GAD7)) - Depression (patient health questionnaire (PHQ9)) - Disease status in CD patients (Harvey-Bradshaw index) - Disease status in UC patients (Simple Clinical Colitis Activity Index) | - UC (56%) - CD (44%) - the mean duration of disease was 5 years | Support needs | Results showed participant accounts illustrate those concerns about underperformance and preventing an accident linked to symptoms of anxiety whilst low mood was associated with a lack of understanding and stigma.  Participants in this study stated a desire for psychological support delivered by a professional with specialized knowledge of IBD to evaluate and build their coping strategies. |
| **Kamp & Brittain, 2018**  **(54)** | N/A | - The purpose of this integrative review is to identify types of treatment and nontreatment decisions and the factors that influence decision-making regarding disease management among individuals with IBD. | Integrative review | Advance search in the database | - psychosocial factors | - N/A | - Information needs - Support needs | Research showed two types of decisions: treatment decisions related to medication and surgery, and no treatment decisions focused on diet modification.  Five themes that influence decisions were identified: experiencing symptoms, provider recommendations, convenience attributes, psychosocial factors, and informational needs.  Most of the studies found a positive relationship between an increased number of symptoms and a patient’s willingness to engage in treatment decisions. |
| **Knowles *et al.,* 2018**  **(53)** | Australia  N= 336  Age mean=37.40 (SD= 11.50)  18.75% male (63)  81.25 % female (273),  advertising via Australian IBD support organizations, IBD-related electronic forums, and social media. | - This study aimed to answer two questions: - To what extent do demographic and disease characteristics predict having a mental health issue?   Of those with a mental health issue, do demographic and/or disease characteristics, and/or degree of psychological distress predict engagement in MHS? | Cross-sectional study | Focus group | - Disease status (MIBDI) - Psychological distress (K10) | - UC (30.95%) - CD (69.05%) | Support needs | The result showed that 52% of participants had a mental health issue and three-quarters reported ongoing symptoms attributed to IBD.  Only a minority accessed mental health support, and the only predictor of this was low income with the level of psychological distress not influencing engagement.  Gastroenterology nurses may need to work with patients to identify and address concerns and perceived barriers to seeking mental health support. |
| **Martin-Fernandez *et al.,* 2018**  **(52)** | Spain  N=420  Age =49.8% were between 20-40 years  30% male (126)  70% female (294),  Spanish Facebook site. | The study aimed to ascertain how patients with inflammatory bowel disease (IBD) felt about the information available and the way that their doctors informed them. | Cross-sectional study | Survey  Questionnaire | - The 39-item survey evaluated the information that the patients had available. - HAD scale for anxiety. | - UC 106 (25.2%) - CD 314 (74.8%) - < 5 years 137 (32.6%) - 5-10 years 93 (22.1%) - 10-20 years 127 (30.2%) - > 20 years 63 (15%) | - Information needs - Support needs | The result of the study showed that patients generally felt that they were well-informed. However, some aspects needed improvements, such as information for younger patients or a lower education level, the information provided at diagnosis, information about specific or insufficiently covered IBD aspects, and suggestions from doctors about high-quality websites. |
| **McDermott *et al.,* 2018**  **(51)** | Ireland  N=322  Age median =38years (29-48),  49% male (159)  51% female (163),  they performed focus groups of patients with IBD and used qualitative analysis to generate hypotheses, then developed a quantitative questionnaire which was disseminated to IBD patients attending 3 different centers. | To assess patients’ education needs in IBD to facilitate the design of a patient education program. | Mixed-method study | Focus Groups  Questionnaire | - Interview for generating hypothesis of patient education in IBD.   A questionnaire focusing on content, medium, and outcomes of a patient education program in IBD. | - UC 135 (43%) - CD 184 (58%) - mean disease duration of 7 years | Information needs | The result of the study showed that patients’ preferences for education include components such as what to expect and diet and patients seem to distrust the internet as an IBD information source. |
| **Philip *et al.,* 2018**  **(50)** | UK  N=136  Age mean = 40years old (18-81)  51% male (70)  49% female (66),  clinics at Hospital | The purpose of the study was to explore patient attitudes towards traveling and to identify areas of unmet need in the IBD service. | Cross-sectional study | Survey  Questionnaire | explores patient attitudes towards traveling to identify areas of unmet need: (questionnaire) | - UC (39%) - CD (54%) - IBDU (5%) - mean disease duration of 12.2 years | - Information needs - Support needs | A considerable number of patients felt that they did not receive adequate medical advice before their travel.  The vast majority of patients were interested in receiving medical advice before their travel abroad which reiterates that the current level of travel advice is insufficient. |
| **Wu & Zhong, 2018**  **(49)** | China  N=159  Age mean =37.1% were between 26–35 years old  71.7% male (114) 28.3% female (45),  outpatient clinic and hospitalization in the Department of Gastroenterology | To explore the demands for different disease-related information and the acceptance of various information sources in patients with CD. | Cross-sectional study | Questionnaire | - Information needs: (questionnaire) | - CD (100%) - Most patients (7, 45.9 %) were diagnosed between 1 to 5 years | Information needs | The disease-related information demands of patients with CD are generally high and are influenced by many variables. Patients living in the countryside and patients with a short disease duration have high information demands concerning life management. Patient acceptance of different information sources is variable. Doctor-mediated guidance, brochures, and videos are the top 3 patient choices for information sources. |
| **Daher *et al.,* 2019**  **(11)** | Israel  N=571  Age mean =34.2 (SD=13.3)  52% male (265)  48% female (240),  hospital-based IBD clinics via email or phone | Identify gaps in the information received, and relate unique patient characteristics to specific information needs. | Observational study | Survey | - Assessing recent disease activity: (Manitoba IBD Index (MIBDI)) - Patient information needs: (questionnaire) | - UC 179 (31.3%) - CD 382 (66.9%) - IBDU 10 (1.8%) - The mean disease duration was 9.7 years for CD and 8.3 years for UC | Information needs | Analysis of various patient profiles revealed associations with specific information topics, paving the way for building patient-tailored information resources |
| **Sarwan *et al.,* 2019**  **(48)** | Spain  N=115  Age median =37years (19-82),  37% male (42)  63% female (73),  Gastrointestinal Unit at the Port of Spain General Hospital | This study aims to determine the psychosocial needs most commonly affected among IBD patients. | Cross-sectional study | Questionnaire | - Psychosocial needs: (Quality of life assessment part of the questionnaire) - Social support: (questionnaire) | - UC 66 (57%) - CD 49 (43%) - median disease duration of 7 years | - Support needs | The top three needs in descending order, were diet, clear-mindedness, and leisure/hobbies.  Results indicated the majority of participants have adequate social support.  No demographic or clinical variables were found to be associated with the participant’s illness perception. |
| **Wåhlin *et al.,* 2019**  **(47)** | Sweden  N=12  Age =41.7% were between 40-49 years old  33.3 % male (4)  66.7% female (8),  gastroenterology unit | The study aimed to explore disease-related worries in persons with CD to gain deeper insight into how this worry is experienced and handled. | Qualitative study | semi-structured interview | - Disease status (HBI) - Anxiety and depression (HADS) - Disease-related worries (semi-structured interview) | - CD (100%) - Most patients (7, 58.3.0%) were diagnosed more than 10 years. | - Information needs - Support needs | This study showed:  Among CD patients with the highest degree of self-reported disease-related worries, the unpredictable course of the disease, impaired function due to fatigue, and lack of control of bowel function are the most prominent causes of worry.  The participants’ worries evoked feelings of stress, guilt, and frustration.  Actively gaining control by changing focus, performing relaxation exercises, and seeking information were found to be important self-care strategies.  A strong and partially unmet need of expressing and venting concerns with a caregiver was identified. |
| **Yu *et al.,* 2019**  **(46)** | China  N=342  Age =41(37-45)  60% male (205)  40% female (137),  8 different hospitals in China | Source and expectations of disease information for Chinese CD patients | Cross-sectional study | - Online questionnaire survey | - Disease status (questionnaire) - IBD-related information needs and sources (questionnaire) - Patients’ expectations for online education (questionnaire)   Medication adherence (Morisky Medication Adherence Scale) | CD (100%) | Information needs | This study showed internet and social media sites such as WeChat have become important sources of information for IBD education in China. The information provided in current WeChat public accounts does not fully meet patients’ expectations. |
| **Casellas *et al.,* 2020**  **(45)** | Spain  N=9,  Confederation Crohn y Colitis Ulcerous (ACCU) is a confederation based on associations of patients with Crohn's disease and UC | Patient preferences in the management of UC. | Qualitative study | - Literature review - The decisions of the nominal group   the Delphi | patient’s preferences: (literature review, the decisions of the nominal group, and the Delphi) | - UC (100%) | - Information needs - Support needs | The following key clinical scenarios were identified: diagnosis, follow-up, surgery, and special situations/patients’ profiles such as adolescents or women.  Patient preferences were classified into information, treatment (pharmacological and not pharmacological), follow-up, relations with health professionals, relations with the health system, and administration.  Finally, 11 recommendations on patient preferences for UC about its management reached the level of agreement established. |
| **Del Hoyo *et al.,* 2020**  **(44)** | Spain  N=18  Age median = 37.5 (20–63),  50% male (9)  50% female (9),  Spanish Confederation of patient associations | this study aimed to adapt the TECCU telemonitoring app to the preferences and needs of IBD patients. | Qualitative study | Focus Groups | - patients´ perceptions to adapt the TECCU telemonitoring app: (Focus group) | - UC 8 (44.5%) - CD 10 (55.5%) - median disease duration of 18 years | Information needs | The patients´ perceptions indicate that platform design should favor shared decision-making with healthcare providers through an open and safe communication process.  The frequency of self-testing was adapted to the participants’ requests, and the app was connected to high-quality and continuously updated web pages. |
| **Feng *et al.,* 2020**  **(43)** | China  N=905  Age = 33.7% were younger than 28 years old  59.3% % male (534)  40.7% female (371),  through www.wjx.cn from six provinces. | To assess the knowledge and vaccination rate of Chinese IBD patients and find the influencing factors. | Retrospective research | Online anonymous questionnaire | - Source of IBD-related vaccine information; and patients’ preferences: (Questionnaire) - Patients’ knowledge of the vaccination: (Questionnaire)   Type of vaccines and disease status: (Questionnaire) | - UC 189 (21%) - CD 693 (77%) - IBDU 18 (2%) - Most patients (657, 73.0%) were diagnosed in less than 5 years. | Information needs | Vaccination-related knowledge levels in IBD patients appeared to be low and the rate of vaccination for both hepatitis and varicella was way below the recommended level. |
| **Karadag *et al.,* 2020**  **(14)** | United Kingdom  N=15  Age mean = 28(20–40)  40 % male (6)  60% female (9),  social media  (Facebook, Twitter and  Instagram.) | Explore patients’ experiences of living with IBD with a focus on their information and support needs. | Qualitative study | Semi-structured  interviews | Patient experiences with a focus on knowledge and information needs (semi-structured interviews) | - UC 8 (53.3%)   CD 7 (46.7%) | - Information need - Support needs | their findings highlight the importance of clear information and support from health professionals, as well as the benefits of online communities for ongoing support. At the point of diagnosis, patients would benefit from information about what IBD is, as well as how it may impact day-to-day life from doctors so social media is not the only source of initial information about IBD. |
| **Keller *et al.,* 2020**  **(42)** | Germany  -  Google trends about IBD. | Identify information gaps to allow a more complete education of patients. | Qualitative study | - Online tracking system | Information gap: (online tracking system) | - N/A | Information needs | This study showed a need for information concerning symptoms, nutrition, and therapy that should be considered during patient education. |
| **Khalil *et al.,* 2020**  **(41)** | USA  N=17 (Focus groups and Interviews)  Focus groups=11  Age median-Focus group = 41 (22-83)  27 % male  73% female  Interviews=6  Age median-Interviews=34 (21-64)  17 % male  83% % female,  Patients with IBD were recruited in three-phase and performed focus groups and interviews. | This study aims to gain an in-depth understanding of the unmet educational needs of patients with IBD through web-based educational videos. | Qualitative study | - Focus groups and   semi-structured phone interviews | - Interview and video testing for the needs of patients with IBD | - UC - CD - Focus groups- median:15 - Interviews median: 12.5 | - Information needs - Support needs | Design thinking offers a deep understanding and recognition of the unmet educational needs of patients with IBD; this approach informed the development of 5 evidence-based educational videos. |
| **Moon *et al.,* 2020**  **(40)** | Korea  N=355  Age mean = 37.6  59.2 % male (210) 40.8% female (145),  30 academic referral hospitals | Investigated the psychosocial burden and factors related to poor health-related quality of life (HRQL) among patients newly diagnosed with moderate-to-severe UC who were affiliated with the nationwide prospective cohort study. | Cohort study | Survey and questionnaire | - psychological distress: Anxiety and depression symptoms (HADS) - Work disability: Work productivity (WPAI)   HRQL: disease-specific (IBDQ), generic (SF-12) | UC (100%),  The mean duration from symptom onset to diagnosis was   - 1. weeks | Support needs | Psychosocial screening and timely interventions should be incorporated into the initial care of patients newly diagnosed with UC. |
| **Santos et al., 2020**  **(10)** | N/A | synthesize the evidence about patients’ perspectives on medication for IBD. | a mixed-method  systematic review | advance search in the database | Patients’ perspectives on medication | - N/A | - Information needs - Support needs | Lack of knowledge, doubts, negative views and misconceptions about medications are some of the perceptions among patients with IBD.  Misconceptions about medication for patients with IBD seem to be fed by the lack of knowledge of the prescription.  Informed patients could lead to better disease management and improved healthcare outcomes.  Health services for IBD could be better planned when taking into consideration patients’ perspectives.  Access to IBD care also needs to be addressed by policymakers |
| **Zare *et al.,* 2020**  **(39)** | Iran  N=26 (patients and health professionals)  Patient=14  Age mean-patients = 29.7 years (19 - 49)  42.8 % male (6)  57.2% % female (8)  Health professionals=12  Age means health professionals=39.6 (25-57)  41.7 % male (5)  58.3% % female (7)  Work experience mean=12.1 (1-30),  Patients were selected from 2 IBD clinics in Tehran and Shiraz cities and Health professionals were selected from different clinics, hospitals, and medical science universities. | The purpose of this study was to identify the dimensions of IBD patients’ empowerment. | Qualitative study | Semi-structured interviews | Dimensions of empowerment in IBD patients | - UC - CD - 6 years | - Information needs - Support needs | The key aspects of the empowerment of IBD patients including self-care, psychological coping, disease-specific health literacy, self-evaluation, and social interaction skills were identified.  The dimensions identified in this study can be used as a basis for educational interventions to enhance patients’ empowerment to prevent the disease from affecting their quality of life negatively. |
| **Al Khoury *et al.,* 2021**  **(4)** | N/A | Assess IBD patient preferences and perspectives during various stages of their disease. | Systematic review | - advance search in the database | - Patient Perspectives and Expectations | - N/A | - Information needs - Support needs | The result of this systematic review indicated that patients with IBD expect more information about their disease process, shared decision-making, and symptom control. |
| **Aluzaite *et al.,* 2021**  **(38)** | UK, Australia, New Zealand, and Israel  N=1878  Age = 48.2% 30–49 years old  (18 - 89 years)  23.7 % male (440)  76.3% female (1431),  social media,  local newspaper advertisements,  patient support organization newsletters and advertisements in IBD clinics | Investigated the IBD-associated travel challenges experienced by the patients and their information-seeking behaviors. | Cross-sectional study | - Combination of online   Paper surveys | - Travel preparation practices: (Online and paper survey)   Information-seeking behavior and perceived barriers: (Online and paper survey) | - UC 670 (35.7%) - CD 1104 (58.8%) - Indeterminate colitis - 53 (2.8%) - IBDU 51 (2.7%) - 49.3% of the study participants have had IBD for more than 10 years | - Information needs - Support needs | The result of this study indicated that travel barriers are very common among IBD patients with consistent characteristics among different countries a global problem that needs to be addressed. |
| **Cai *et al.,* 2021**  **(1)** | N/A | Focus on the potential methods for the treatment of IBD, providing a comprehensive overview for clinicians of available therapies and drugs for IBD treatment. | Comprehensive review | advance search in the database | treatment of IBD | N/A | - Information needs - Support needs | Advances in the management of IBD have led to a paradigm shift in the treatment goals, from targeting symptom-free daily life to shooting for mucosal healing.  The treatment of IBD is primarily pharmacological.  New therapies for IBD treatment mainly include apheresis therapy, improvement of intestinal microecology, stem cell transplantation, and exosome therapy.  Timely communication and close cooperation between doctors and patients are equally essential to effective treatment strategies.  Endoscopy, histology, radiology, immune biochemical monitoring biomarkers, quality of life assessment, and other methods have been introduced to provide more valuable references for the assessment of disease activity. |
| **Chan *et al.,* 2021**  **(37)** | N/A | to comprehensively identify the vaccination rates among IBD patients, predictors of vaccination, reasons for vaccine hesitancy and acceptance, and to assess the outcomes of interventions. | Systematic review | advance search in the database | Vaccination rates, predictors of vaccination, reasons for vaccination hesitancy and acceptance, and outcomes of intervention. | - N/A | - Information needs - Support needs | The vaccination rates of IBD patients, similar to other chronic diseases, are suboptimal. Lack of vaccine-related knowledge and awareness of the need for vaccination are the main reasons for vaccine hesitancy amongst patients and physicians. |
| **Fawson *et al.,* 2021**  **(2)** | UK  N=40  Age = (23 - 60 years)  45 % male (18)  55 % female (22),  they recruited from clinics in three hospitals across northern and southern England and online from members of the UK national Crohn’s and Colitis charity. | Understand patients’ symptom self-management strategies and preferred design for a future online symptom self-management intervention. | Qualitative study | a focus group (framework analysis)  Individual interviews (thematic analysis) | Daily experiences: (focus groups)  Patients’ self-management strategies: (Interview) | - UC 17 (42.5%) - CD 21 (52.5%) - IBDU 2 (5%) - The mean disease duration was 14.9 years | - Information needs - Support needs | The result of this study indicated that patients had numerous ways of self-managing symptoms of fatigue, pain, and urgency/incontinence related to IBD and expressed their needs for the content, design, and functionality of the proposed intervention. |
| **Goodsall *et al.,* 2021**  **(36)** | Australia  N=97  Age median=49 years (17-77),  IBD database of patients known to the  Hospital IBD unit | Evaluate the attitudes, concerns, and health behavior of IBD patients during COVID‐19. | Cross-sectional study | Online survey | Perceived risk of COVID19: (Questionnaire) | - UC 38 (39%) - CD 57 (59%) - Unsure/indeterminate 2 (2%) | - Information needs - Support needs | The result of the study indicated that most participants would like to receive more information about the current understanding of how IBD medications may affect COVID‐19 risk. |
| **Graffigna *et al.,* 2021**  **(9)** | USA  N= 73 | Define the psychosocial needs of IBD patients and promote their engagement in daily clinical practice. | Consensus conference | N/A | - Main strategies to ensure the management of the psycho‑social needs of pediatric IBD patients to improve their patient engagement | - N/A | - Information needs - Support needs | It is necessary for early detection of and, in case of need, the intervention of the psychosocial needs of patients to achieve patient involvement in IBD care. |
| **Kutschera *et al.,* 2021**  **(35)** | Austria  N=1286  Age mean =40  52.2 % male (671)  47.8 % female (615),  IBD outpatient clinics outside  university hospitals | This study investigated patients’ subjective need for integrated psychosomatic support and psychotherapy and indicators for it. | Cross-sectional study | Survey | - Patients subjective need a Short IBD Questionnaire (SIBDQ) for assessing their quality-of-life IBD patients - Assessment of the Demand for Additional Psychological Treatment Questionnaire (ADAPT) - The international questionnaire to measure the use of complementary and alternative medicine in German (ICAM-G). | - UC 435 (%33.8) - CD 830 (%64.5) - IBDU 21 (%1.6) - median=10 years | - Support needs | The result of this study concluded that patients with IBD have a high need for either integrated psychosomatic support, psychotherapy, or both. |
| **Long *et al.,* 2021**  **(34)** | USA  Recruitment was conducted via social media  efforts of the Crohn’s & Colitis Foundation | experiences of IBD patients during the COVID-19 pandemic.  Prioritize patients’ information needs and preferences. | Qualitative study | Semi-structured virtual focus groups | - IBD patient experiences and concerns | - UC - CD | - Information needs - Support needs | Information needs for patients during the COVID-19 pandemic centered upon understanding disease-specific risks and Identifying challenges and fears will inform future research agendas and communication with patients. |
| **Popov *et al.,* 2021**  **(33)** | Canada  N=17  Age mean =43 (25 - 77)  53 % male (9)  47 % female (8),  Gastroenterology Clinic | Explore the various challenges patients  encounter in living with IBD and to propose suggestions for overcoming them | Qualitative study | Focus group (A semi-structured interview) | - Patients’ Experiences and Challenges: (Semi-structured interview). - Awareness of Psychosocial impacts on the financial burden on quality of care. | - UC 2 (%11.8) - CD 15 (%88.2) - The mean disease duration was 15 years | Support needs | Family-based education approaches, psychotherapy, education, awareness addressed to the aforementioned stakeholders, and expansion of community support groups are required for improving overall IBD care. |
| **Rubin *et al.,* 2021**  **(32)** | Australia, Canada  Finland, France  Germany, Italy  Japan, Spain  UK, and USA  N=3354 (2100 patients and 1254 physicians)  Age means patient =40.8  53 % male (1113)  47% female (987)  Age means physicians =47.6  53 % male (1113)  47% female (987),  Patients were recruited from databases | Examined patient and physician perspectives on living with UC and tried to identify gaps in optimal care. | Narrative Global Survey | Questionnaire | - Patient-physician communication: (survey) - Knowledge of UC: (survey)   Information and support: (survey) | - UC (100%) - mean of 8.8 years | Support needs | These survey results highlight overall patient satisfaction with patient-physician communication but emphasize areas for improvement, such as patients' desire to have more information earlier in their disease course. There is an unmet need for better information, materials, and support. Physicians need to consider which of the available tools and resources can help patients talk more openly, and accurately because informed patients are more likely to engage with physicians in a shared decision-making process. |
| **Volpato *et al.,* 2021**  **(5)** | N/A | Identifying the key concepts that characterize the psychological, social care, and welfare needs of people affected by IBD across the lifecycle by highlighting the main types of published evidence and sources available.  Explore previous research activity, disseminate findings, and identify gaps in the comprehension of patients’ needs. and explore factors that increase patient engagement | Scoping review | advance search in the database | perceived engagement and care needs | - N/A | - Information needs - Support needs | The literature showed the relevance of organizing a flexible, personalized healthcare process across all the critical phases of the life cycle, providing adequate benchmarks for comparison from a multidisciplinary perspective and ensuring continuity between hospital and territory. |
| **Vutcovici *et al.,* 2021**  **(31)** | Canada  N=26  Age mean =41.3  34.6 % male  65.4% female,  Crohn’s and Colitis Canada database  of IBD patients | Identify patient perspectives amenable for conversion into measurable IBD care quality indicators. | Qualitative study | - Focus groups | - Patient's perspective on IBD care services (Focus group) | - UC - CD - 65% were living with their disease for more than 5 years | Support needs | Several perceived unmet needs were elicited from participants that could be converted into measurable quality indicators.  These unmet needs addressed the need for information, access to multi  Disciplinary services and specialized care, and access to psychological support.  Patient unmet needs informed the selection of nine quality indicators that were included in the final list of PACE indicators to assess IBD care services across Canada. |
| **Goren *et al.,* 2022**  **(30)** | Israeli  N=534  Age median =38 years (28.7-51.0)  45 % male (229)  42.8 % female (305),  social media | The aim was to assess topics of interest and concerns and identify information gaps among patients with IBD who are active on social media. | Mixed-method study | - Structured online survey - Online survey and thematic analysis | - satisfaction with the HCP-guided SM network (online survey) - Information preferences: (online survey) | - UC - CD - the median duration of the disease was 10 years | Information needs | They found that patients with IBD who were active on an HCP-guided SM network were satisfied with the platform. Respondents with IBD who were active online exhibited a substantial disease burden and a relatively complicated disease course, as reflected by the high proportion of biological therapy use and care received at referral IBD centers.  They identified 18 distinctive topics of interest, of which the top 5 rated were diet, lifestyle, CAM, interpretation of diagnostic tests, and specialist referrals and reviews. |

**IBD:** Inflammatory bowel disease; **IBS:** Irritable bowel syndrome; **UC:** Ulcerative colitis; **CD:** Crohn’s disease; **IBDU:** Inflammatory bowel disease unclassified; **RCT:** Randomized control trial
